# Direct comparison of *Arabidopsis* gene expression reveals different responses to melatonin versus auxin

**DOI:** 10.1186/s12870-019-2158-3

**Published:** 2019-12-19

**Authors:** Sajal F. Zia, Oliver Berkowitz, Frank Bedon, James Whelan, Ashley E. Franks, Kim M. Plummer

**Affiliations:** 1 Department of Animal, Plant and Soil Sciences, AgriBio, La Trobe University, Bundoora, VIC 3086 Australia; 2 ARC Centre of Excellence in Plant Energy Biology, La Trobe University, Bundoora, VIC 3086 Australia; 3 Department of Physiology, Anatomy and Microbiology, La Trobe University, Bundoora, VIC 3086 Australia; 4 Centre for Future Landscapes, La Trobe University, Bundoora, VIC 3086 Australia

**Keywords:** Melatonin, Auxin, Promoter activation, Transcriptome, *Arabidopsis thaliana*

## Abstract

**Background:**

Melatonin (*N*-acetyl-5-methoxytryptamine) in plants, regulates shoot and root growth and alleviates environmental stresses. Melatonin and the phyto-hormone auxin are tryptophan-derived compounds. However, it largely remains controversial as to whether melatonin and auxin act through similar or overlapping signalling and regulatory pathways.

**Results:**

Here, we have used a promoter-activation study to demonstrate that, unlike auxin (1-naphthalene acetic acid, NAA), melatonin neither induces *Direct repeat 5 DR5* expression in *Arabidopsis thaliana* roots under normal growth conditions nor suppresses the induction of *Alternative oxidase 1a AOX1a* in leaves upon Antimycin A treatment, both of which are the hallmarks of auxin action. Additionally, comparative global transcriptome analysis conducted on Arabidopsis treated with melatonin or NAA revealed differences in the number and types of differentially expressed genes. Auxin (4.5 μM) altered the expression of a diverse and large number of genes whereas melatonin at 5 μM had no significant effect but melatonin at 100 μM had a modest effect on transcriptome compared to solvent-treated control. Interestingly, the prominent category of genes differentially expressed upon exposure to melatonin trended towards biotic stress defence pathways while downregulation of key genes related to photosynthesis was observed.

**Conclusion:**

Together these findings indicate that though they are both indolic compounds, melatonin and auxin act through different pathways to alter gene expression in *Arabidopsis thaliana*. Furthermore, it appears that effects of melatonin enable *Arabidopsis thaliana* to prioritize biotic stress defence signalling rather than growth. These findings clear the current confusion in the literature regarding the relationship of melatonin and auxin and also have greater implications of utilizing melatonin for improved plant protection.

## Background

Plant hormones are considered as major determinants of plant’s overall growth and development. Multiple plant hormones such as auxin, cytokinin (CK), gibberellins (GA) and brassinosteroids (BR) have been shown to exert key functions in regulating various developmental processes such as, seed and fruit development, shoot and root architecture [1]. With the advancement in forward and reverse genetics, there is now a good understanding of how these hormones are perceived and the key players identified in their signalling pathways. For this purpose, plant hormones and their signalling transduction networks have been widely studied and employed to improve sustainable agriculture such as stem elongation, flowering time and processes like nitrogen use efficiency [2]. Interestingly, these effects on growth regulation are controlled by interacting signalling pathways among plant hormones. This interaction is either antagonistic, synergistic or occurs in parallel [3]. For example, processes such as seed germination, shoot and root growth and grain-filling are governed by antagonistic relationship between abscisic acid (ABA) and ethylene (ET) in maize [4]. Moreover, auxin and cytokinin exhibit antagonism during formation of root apical meristem but act in synergy during shoot apical meristem formation [5].

Melatonin (MT) is an indolic molecule ubiquitously present in all living organisms. Melatonin in plants is associated with growth and development, such as leaf and root organogenesis, senescence and flowering [6–9]. Moreover, melatonin also mitigates a variety of abiotic and biotic stresses in plants such as cold, drought, heat and infections by the  fungal pathogen *Diplocarpon mali*, biotrophic and hemibiotrophic bacteria *Xanthomonas oryzae* and *Pseudomonas syringae* DC3000, respectively [10–15]. To date, melatonin is considered as a growth-regulating secondary metabolite in plants but recent studies suggest that it also has the potential to be a plant hormone [16]. In order to be considered as potential plant hormone, there are certain fundamental characteristics that a candidate molecule needs to exhibit. These include knowledge about biosynthetic pathway, receptor and physiological effects. Studies on biosynthetic pathway of melatonin in plants have made considerable progress. These show tryptophan (Trp) as the precursor followed by four sequential reactions with enzymes Tryptophan decarboxylase (TDC), Tryptophan-5-hydroxylase (T5H), Serotonin-*N*-acetyltransferase (SNAT) and Acetyl serotonin methyltransferase (ASMT) [17, 18]. This has been proposed to be the standard biosynthetic route in plants such as *Arabidopsis thaliana* and Rice *(Zea mays)* under normal growth conditions, however, an alternate pathway has also been proposed to exist under conditions such as senescence in which the key enzymes SNAT and ASMT switch in their order to produce melatonin. This has also been proposed to be the most prevailing biosynthetic route in *Arabidopsis thaliana* and Rice *(Zea mays)* as compared to the classic route [19]. Recently, a reverse biosynthetic reaction involving an enzyme *N*-acetylserotonin deacetylase (ASDAC) has been identified in *Arabidopsis thaliana* and rice in which melatonin intermediate *N*-acetylserotonin is rapidly converted to serotonin. This reaction restricts synthesis of melatonin thereby maintaining optimal levels of melatonin for balanced plant growth and development [20]. Like other plant hormones, melatonin exerts multiple physiological effects in plants such as regulation of stomatal opening/closing, photosynthesis, tropism, changes in metabolism of carbohydrates and nitrogen and cellular effects like changing the intracellular calcium (Ca^2+^) content and membrane permeability [21–24]. Recently, the first melatonin receptor CAND2/PMTR1, a G-protein coupled receptor has been identified in *Arabidopsis thaliana* which was shown to regulate stomatal closure mediated by melatonin [25]. This was long-sought because a lack of melatonin receptor in plants was impeding the full understanding of melatonin-mediated signalling. Without an identified receptor, it was also a challenge to view melatonin as a potential plant hormone.

Recent studies have investigated the crosstalk of melatonin with the well-known plant hormones such as salicylic acid (SA), abscisic acid (ABA), and ethylene [26]. Of particular interest has been the comparison between melatonin and the widely studied hormone, auxin, because of their common biosynthetic precursor, tryptophan, which leads to structural similarities such as having an indole core. These similarities have led to the hypothesis that melatonin could also share auxin-like activities, in terms of regulating growth in a concentration-dependent manner. However, the current understanding of the relationship between melatonin and auxin remains unclear. Previous studies using promoter-reporter constructs, gene expression and physiological responses both support and contradict similar modes of action or overlapping signalling pathway between auxin and melatonin. In support of similar functions, it has been reported that melatonin stimulated plant growth at low concentrations (10^− 4^ M,10^− 7^ M, 0.01 M) similar to auxin by increasing root growth, lateral and adventitious root formation in a variety of plant species [6, 27, 28]. Similarly, in roots of *Brassica juncea*, melatonin treatment (0.1 μM) increased the concentration of indole-acetic acid (IAA) and enhanced root growth [13, 29]. In, transgenic tomato plants over-expressing the ovine melatonin biosynthetic pathway gene, *Serotonin-N-acetyltransferase* (*SNAT),* led to a decrease in IAA levels and loss of apical dominance [15, 30]. Similarly, melatonin decreased the transcript abundance of *YUCCA (YUC)* (*YUC1, YUC2, YUC5, YUC6* and *TAR2*) auxin biosynthetic genes upon 600 μM treatment in Arabidopsis roots [31]. Auxin-responsive marker lines such as *Direct Repeat 5, DR5,* have been used to investigate the response and distribution of auxin in many plant species such as Arabidopsis and soybean [32]. *DR5* is a synthetic promoter containing auxin responsive elements (AuxREs) and is widely utilized as an indirect marker of endogenous auxin distribution, signalling and responses [33, 34]. Wang and colleagues showed that exogenous melatonin treatment at an inhibitory concentration (600 μM) to Arabidopsis roots enhanced *GFP* and *GUS* expression of *DR5* lines [31]. Moreover, RNA-sequencing analysis from 10 and 20 μM melatonin-treated roots of 2-week old rice seedlings showed that auxin-signalling genes were significantly increased in abundance [35]. In contradiction to above studies, Kim et al. (2016) reported the inability of melatonin (10^− 7^ M–10^− 4^ M) to stimulate the plant responses in maize in the classical bioassays that are specifically used to demonstrate auxin responsiveness i.e. elongation of coleoptiles, inhibition of roots in young seedlings and induction of ethylene biosynthetic gene, *1-aminocyclopropane-1-carboxylate (ACC) synthase* [36]. Gene expression studies in Arabidopsis plants treated with 100 pM and 1 mM melatonin revealed that auxin biosynthetic and related genes were not changed in transcript abundance except for one auxin-responsive gene *IAA-amino synthase,* that was increased in abundance upon melatonin treatment [37]. It has been shown that melatonin treatment (5, 100, 450 and 500 μM) was unable to induce expression of auxin-responsive marker line *DR5:GUS* in *Arabidopsis* seedlings [38, 39]. The data from these studies point to the contrasting findings between and within plant species. A common confounding factor especially for transcriptomic analysis, has been the lack of direct comparisons between melatonin and auxin treatments under identical set of experimental conditions.

Interplay of melatonin and mitochondria has been extensively studied in mammals but just recently begun to be investigated in plants [40, 41]. Mitochondria are the powerhouses of cells and play a key role in growth and development of plants by providing necessary metabolites, enzyme cofactors and energy (ATP). Recent studies have shown that mitochondria play integral role in cellular signalling. Mitochondrial signalling, or mitochondrial retrograde signalling results when mitochondria functioning is perturbed by stimuli and this leads to transmission of signals to alter nuclear gene expression [42]. This shows that mitochondria are not only crucial for plant’s growth and development but also for driving responses to biotic and abiotic stresses. It is thus not a surprise that there exists an interaction between mitochondrial and hormone signalling pathways as hormones are strongly linked with processes of growth and stress defence [43]. Nuclear genes encoding mitochondrial proteins have been shown to be responsive to a variety of hormone treatments based on a meta-analysis study. The main regulators of mitochondrial function identified were plant hormones auxin, cytokinin (CK), jasmonic acid (JA), and salicylic acid (SA) [43]. More direct targeted approaches have shown an interaction between these hormones and mitochondrial signalling. For example, ABA-induced signalling of guard cells in response to drought stress is negatively regulated by a pyruvate carrier of mitochondria termed as *Negative Regulator of Guard Cell ABA Signalling 1,* (*NRGA1)* in *Arabidopsis thaliana* [44]. Salicylic acid (SA) treatment has been shown to uncouple and inhibit mitochondrial electron transport in *Nicotiana tabacum* [45]. Auxin and mitochondrial respiration has been long hypothesized to have a connection [46]. Moreover, multiple studies have shown a link between auxin responses and mitochondrial function [47, 48]. The mutants of genes encoding proteins that are involved in the synthesis of the inner mitochondrial membrane such as Filamentation Temperature Sensitive H 4 (FTSH4) and PROHIBITIN3 were shown to inhibit auxin response [49, 50]. Additionally, auxin was oxidatively degraded in the *ftsh4* mutant through hydrogen peroxide (H_2_O_2_)-mediation which is suggested to be a strategy to prioritize processes such as stress defence over growth-related processes [51]. Antimycin A is a chemical stimulus of mitochondrial stress that acts by blocking complex III of the mitochondrial respiratory chain. Treatment by antimycin A also led to decreased auxin (IAA) levels and down-regulation of auxin receptors and transporters such as auxin efflux transporters PIN1/3/4/7 in *Arabidopsis thaliana* [52, 53]. Additionally, auxin homeostasis was defective in mutants of the gene *IAA-alanine Resistant 4 (IAR4)* which encodes a putative mitochondrial pyruvate dehydrogenase E1 alpha-subunit suggesting its integral role in maintaining auxin homeostasis [54].

Alternative oxidase (AOX) is a terminal oxidase which is part of the plant mitochondrial electron transport chain and acts to uncouple respiration by bypassing proton-pumping complexes III and IV, thereby reducing excessive burst of reactive oxygen species (ROS). This activity is particularly dominant under stressful environmental conditions such as drought, low temperature and bacterial infection by *Pseudomonas syringae* where studies have shown a remarkable increase in AOX transcript and/or protein [55]. This indicates that a wide array of pathways can trigger AOX and hence it is considered as a marker for mitochondrial retrograde signalling. While a range of plant hormones can trigger/induce AOX such as SA and ET [45, 56] others such as auxin are antagonistic to the induction of AOX [52]. Auxin (4.5 μM NAA) application was shown to inhibit the Antimycin A-mediated induction of promoter-reporter *Alternative oxidase1a (AOX1a::LUC)* in Arabidopsis [52]. The antagonistic relationship of auxin and mitochondrial retrograde signalling plays a central role in balancing growth and stress responses. Mitochondria along with chloroplasts have been hypothesized to be the original sites of synthesis of melatonin. This relates to the endosymbiotic theory where these organelles are considered to be the descendants of endosymbiotic bacteria which produced melatonin [57]. Very recently, synthesis of melatonin in mitochondria, as well as chloroplasts has been reported in leaves of apple *Malus zumi* and Arabidopsis. Moreover, apple melatonin biosynthetic genes *Serotonin N-acetyltransferase SNAT* and *Acetylserotonin O-methyltransferase ASMT* were found to be localized to mitochondria and chloroplasts, respectively in both apple and Arabidopsis [41, 58]. However, there is lack of understanding in how melatonin functions with mitochondrial retrograde signalling and its relatedness with auxin. Thus, alternative oxidase is an ideal marker to test the interaction between auxin and melatonin.

In this study, the effects of melatonin were compared directly to auxin treatments. Two different transgenic Arabidopsis lines carrying inducible promoter-reporter constructs that are responsive to auxin were used to compare the response of plants to melatonin versus auxin. *Direct repeat 5 (green fluorescent protein*) *DR5::GFP* as a marker for auxin response and *Alternative oxidase1a (luciferase) AOX1a::LUC* as a marker for mitochondrial retrograde signalling was used [52, 59]. Furthermore, the potential molecular crosstalk between melatonin and auxin was investigated using global transcriptome analysis of Arabidopsis rosette leaves of seedlings whose roots were treated with either melatonin or auxin.

## Results

### Effect of melatonin on *DR5::GFP* expression in primary root tips

**Fig. 1:**
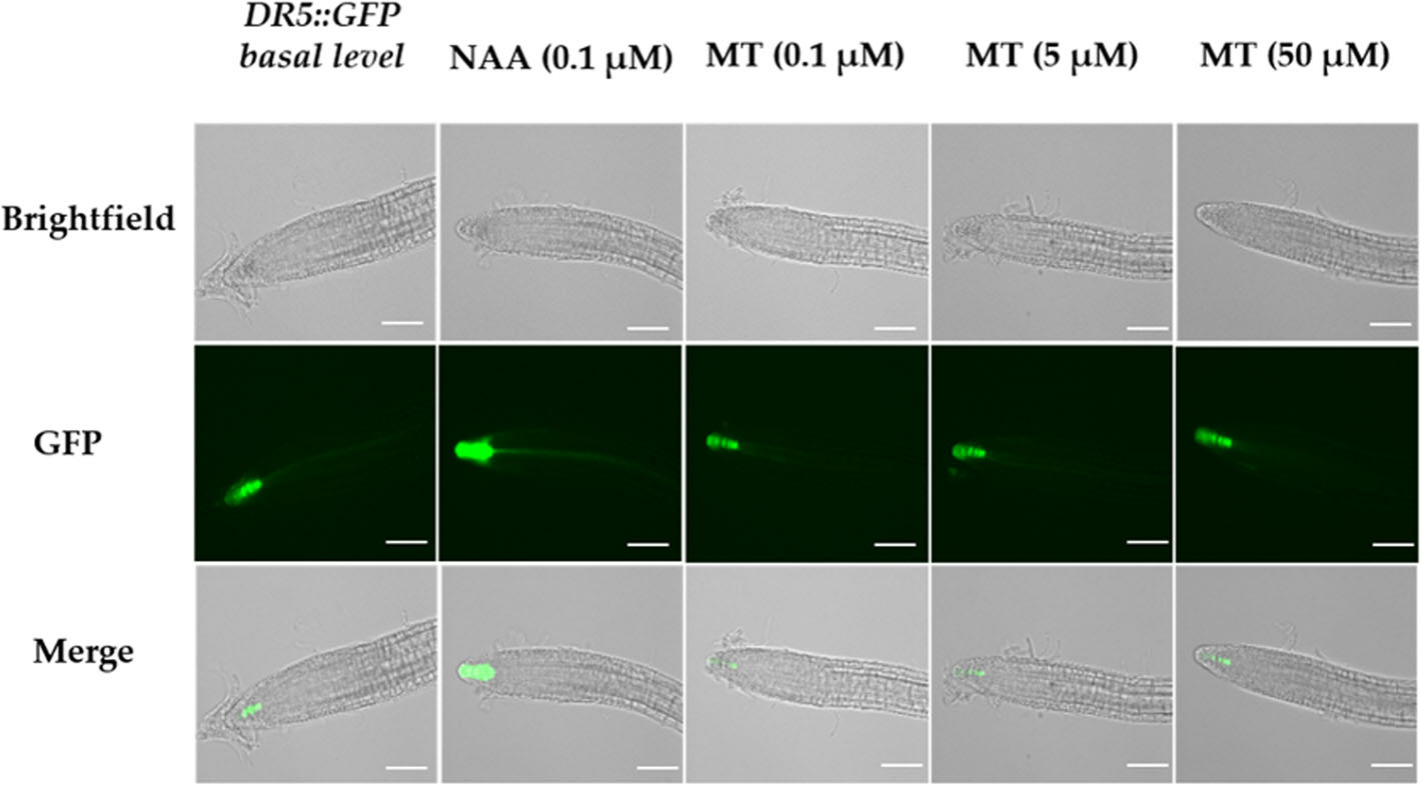
Effect of MT on *DR5::GFP* expression in 5-day old primary root tip of *Arabidopsis thaliana*. The solvent control (0.1% v/v ethanol) represents the basal level of *DR5::GFP* expression. Figure shows representative images of three biological repeats conducted on independent days with ten plants per treatment per replicate. Scale bar = 100 μm, exposure level = 3.5 ms and magnification = 20X

The *DR5*::*GFP* auxin-responsive marker line was used to assess whether melatonin (MT) and NAA treatments have similar effects on *DR5* transactivation in primary root tips of 5-day old *Arabidopsis thaliana* seedlings. Arabidopsis *DR5::GFP* root tips grown on half strength MS media or solvent control (0.1% v/v ethanol) for 5 days, showed basal GFP fluorescence indicative of *DR5* expression at the columella cells of the primary root tip (Fig. 1). When roots were treated with 0.1 μM NAA (used as a positive control), the GFP expression was more intense compared to basal expression as expected and reported by previous studies [32, 60]. Addition of melatonin to concentrations of 0.1, 5 and 50 μM in the growth media did not affect the expression of *DR5*:*:GFP* in primary root tips in terms of intensity of location, neither enhancing nor suppressing the GFP expression. GFP fluorescence intensity was also quantified by calculating Integrated Density (sum of pixel) (Additional file 1: Figure S1). NAA (0.1 μM) treatment enhanced *DR5* transactivation by 2.5-fold compared to solvent control (0.1% v/v ethanol) whereas melatonin treatments up to 50 μM were similar to the basal GFP level expression (Additional file 1: Figure S1). Cellular localization of *DR5* at the columella cells of the root cap was however same regardless of all treatments.

### Effect of melatonin on *AOX1a::LUC* expression in leaves treated with Antimycin A

**Fig. 2:**
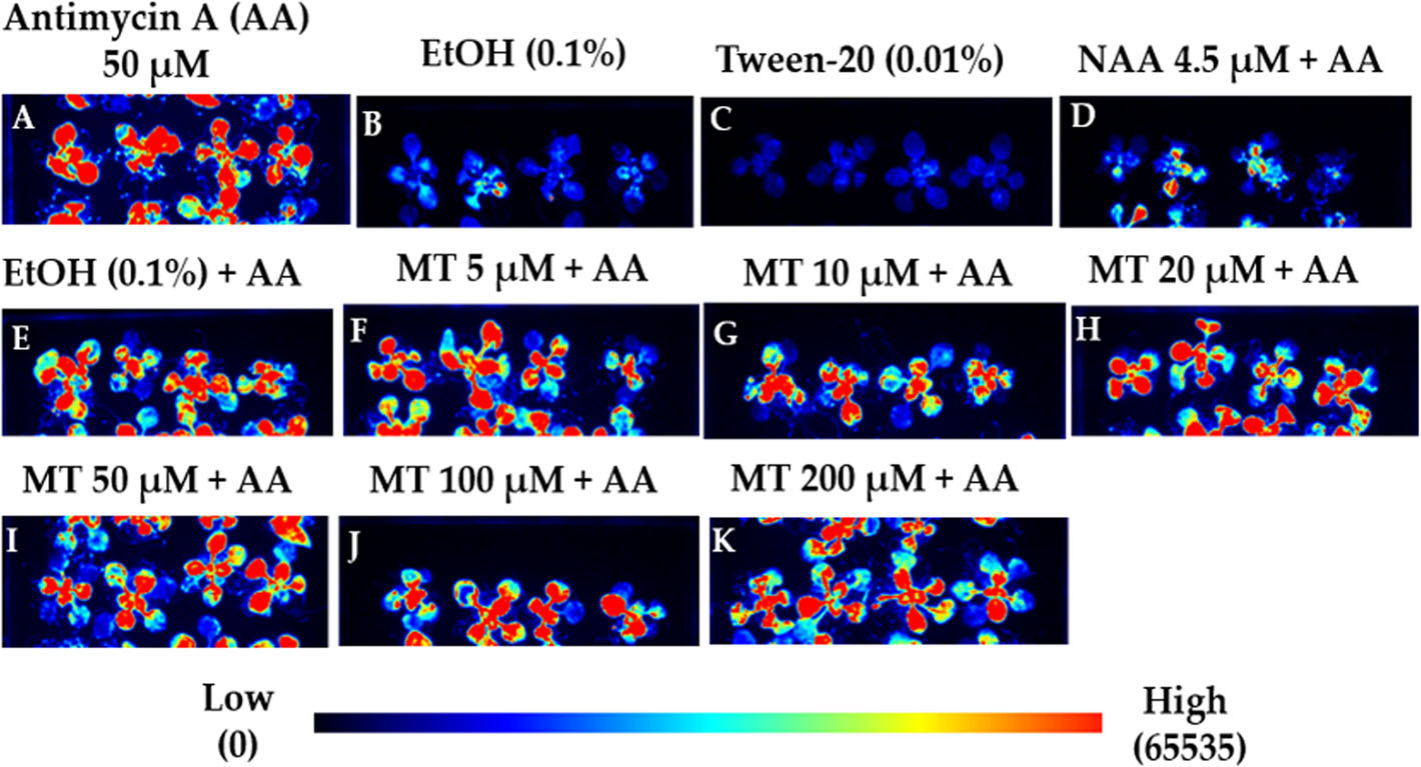
Effect of MT on *AOX1a::LUC* reporter expression in rosette leaves treated with Antimycin A (AA). Bioluminescence images of *AOX1a::LUC* reporter (Col-0) plants grown on +/− MT or NAA containing media for 3 days and sprayed with +/− AA and visualized for *LUC* activity after 6 h in ChemiDoc (BioRad). (**a)** AA spray (**b**) Solvent control (ethanol 0.1% v/v) for AA spray (**c**) Spray surfactant control (0.01% tween-20 v/v) (**d**) NAA-supplemented media and plants sprayed with AA (**e**) media supplemented with solvent control (0.1% ethanol v/v) for MT and NAA and plants sprayed with AA (**f**–**k**) MT-supplemented media and plants sprayed with AA. Figure shows representative images of three biological repeats conducted on independent days with 12 plants per treatment per replicate. All the images correspond to the *AOX1a::LUC* reporter

An *Arabidopsis thaliana* transgenic line carrying a promoter-reporter *AOX1a::LUC* construct [61, 62] was used to assess whether melatonin could, like auxin, negatively regulate the expression of *AOX1a* under 50 μM Antimycin A spray treatment. The enhanced expression of *AOX1a::LUC* induced by Antimycin A was significantly suppressed when plants were pre-treated with NAA treatment, bringing LUC luminescence down to the basal expression level (Fig. 2) as previously reported [52]. The melatonin treatments (5 μM, 10 μM, 20 μM, 50 μM, 100 μM and 200 μM) were chosen based on previous literature studies where 10–100 μM has been reported to affect gene expression in Arabidopsis [10, 12] and an approximate equimolar concentration of melatonin (5 μM) and NAA (4.5 μM) were also used. None of the melatonin treatments suppressed *AOX1a* expression under Antimycin A treatment compared to NAA treatment. By also quantifying luminescence intensity through the integrated density method (ImageJ), it was observed that melatonin (5 μM, 20 μM, 50 μM, and 200 μM) did not significantly suppress *AOX1a* expression as compared to the solvent control (0.1% v/v ethanol) (Additional file 1: Figure S2A). Melatonin (at 10 and 100 μM) slightly reduced *AOX1a* transactivation compared to control treatment but expression level was still significantly higher (3-fold) than the levels at NAA treatment (Additional file 1: Figure S2A). Melatonin or NAA treatments alone were not statistically different to the solvent-only control (0.1% v/v ethanol) (Additional file 1: Figure S2 B, C). Overall, it can be concluded that melatonin does not suppress the induction of *AOX1a* by Antimycin A, in contrast to NAA which suppresses this induction.

### Analysis of differentially expressed genes (DEGs) in leaves of melatonin and NAA treated seedlings

**Fig. 3:**
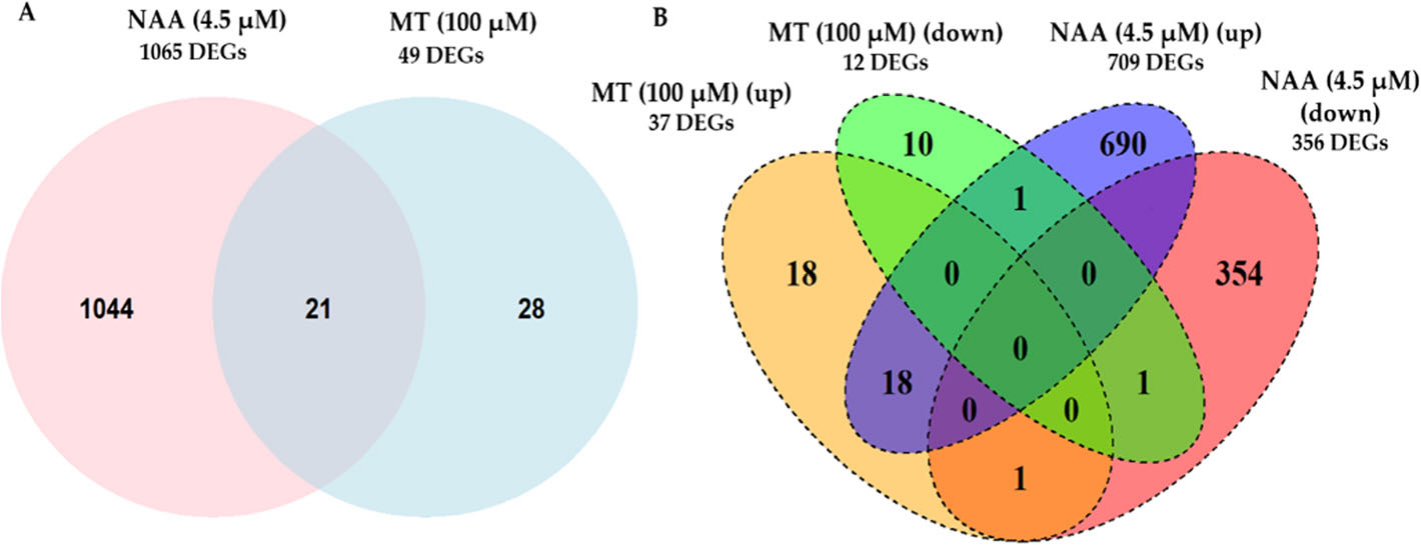
Expression patterns of DEGs in 15-days old rosette leaves in response to MT or NAA (**a**) Venn diagram indicating the common and exclusive DEGs in response to MT (100 μM) or NAA (4.5 μM) as compared to the untreated control (0.1% v/v ethanol). (**b**) Quad Venn diagram showing the number of up- and down-regulated genes in exclusive and common DEGs. The numbers shown in each set indicate the number of genes regulated in response to MT and/or NAA. Genes were called as differentially expressed with FDR < 0.05 and |log2fold change| ≥ 1.2. Three independent biological experiments were conducted for the analysis

Given the differential responses of melatonin and NAA toward auxin-signalling pathways, we compared the transcriptome responses of Arabidopsis leaves from seedlings supplemented with melatonin (5 μM or 100 μM) or NAA (4.5 μM) to gain further insights into the modes of action of these indoles. Differentially expressed genes (DEGs) in melatonin (5 or 100 μM) or NAA (4.5 μM) treated samples were obtained by comparison with their respective untreated solvent controls (0.1% v/v ethanol). A false discovery rate of FDR < 0.05 and log_2_ fold change (log_2_ FC) ≥1.2 were utilised to call transcripts as significantly differentially expressed. No statistically significantly different gene expression was observed upon treatment with melatonin at 5 μM. However, there were remarkable differences observed in the number of genes significantly expressed between melatonin (100 μM) and NAA (4.5 μM) treatment as compared to the untreated solvent control (0.1% v/v ethanol). NAA had a greater impact on gene expression, with a total of 1065 genes significantly differentially expressed, whereas 100 μM melatonin had a modest effect on transcriptome, with only 49 genes differentially expressed (Fig. 3a). Comparison of DEGs differentially expressed upon exposure to either NAA (specifically auxin-responsive genes) or melatonin (100 μM) in our study with other published transcriptome data sets revealed approximately 30–40% overlap [37, 63, 64]. It is important to note that there were differences between our study and previous transcriptome studies in terms of type of auxin used, concentrations, tissue, exposure time and development stage analysed (Additional file 1: Table S1 and S2).

Both treatments shared a set of 21 differentially expressed genes (DEGs). To further obtain statistical insight, a hypergeometric test was employed with ‘dhyper’ command in R, to analyse the significance of overlap, relative to the genomic background between two gene lists and thus assesses the likelihood that any overlap between DEGs of gene lists is non-random [65–67]. It was found that the overlap between DEGs upon exposure to melatonin and auxin was statistically significant at a *p-*value of 2.2 X 10^− 17^.

Transcript levels of a total of 709 genes were significantly enhanced in abundance, and 356 genes significantly decreased in abundance, in response to NAA treatment (Fig. 3b). In contrast, transcript abundance of only 37 genes were significantly increased, and 12 genes significantly decreased in abundance, in response to melatonin (100 μM) treatment. Among these, the expression of 28 genes was uniquely altered by melatonin, 18 of which were enhanced, and 10 decreased in expression (Table 1). In contrast, a total of 1044 genes were exclusively differentially expressed by NAA, with 690 genes showing increased expression and 354 genes with decreased expression. Among the 28 DEGs exclusive to melatonin treatment, 18 genes having increased expression are annotated as being defensins or defensin-like (five), transcription factors (four) and being involved in post-translational protein modification (three), while most of the 12 genes with decreased expression were assigned to being involved in photosynthesis (six) (Table 1). Transcript levels of genes exclusively altered by auxin are listed in detail in Additional file 2: Table S4. These included pathways reported by other studies such as root development and auxin-signalling genes such as transport, biosynthesis and conjugation [63, 64, 68].

**Table 1:** DEGs exclusively regulated by MT (100 μM) as compared to control

Gene ID	Annotation	MT (100 μM)	
		FDR^a^	Log2 (Fold Change)
*Defensins (5)*			
*AT3G59930*	*Defensin-like (DEFL) family protein*	6.0E-04	4.92
*AT5G33355*	*Defensin-like (DEFL) family protein*	6.0E-04	4.92
*AT1G34047*	*Defensin-like (DEFL) family protein*	7.7E-03	4.88
*AT2G26010*	*Plant defensin PDF1.3*	1.2E-02	4.62
*AT2G26020*	*Plant defensin PDF1.2b*	2.7E-02	4.33
*Transcription factors (5)*			
*AT2G47950*	*Myelin-transcription factor like protein*	1.7E-02	4.11
*AT1G06160*	*Ethylene responsive factor AP2/ERF59*	7.7E-03	2.47
*AT3G51910*	*Heat shock transcription factor HSFA7A*	2.4E-02	1.52
*AT5G07100*	*WRKY DNA binding protein WRKY26*	2.5E-03	1.23
*AT5G17300*	*Myb-like transcription factor RVE1*	3.3E-02	−1.42
*Photosynthesis (7)*			
*ATCG00330*	*Chloroplast ribosomal protein RPS14*	5.9E-06	−1.55
*ATCG00020*	*Photosystem II reaction center A PSBA*	1.9E-03	−1.49
*ATCG00350*	*Photosystem I, PsaA/PsaB*	8.9E-10	−1.47
*ATCG00280*	*Photosystem II reaction center C, PSBC*	1.9E-04	−1.43
*ATCG00340*	*Photosystem I, PsaA/PsaB*	1.8E-13	−1.41
*ATCG00270*	*Photosystem II reaction center D PSBD*	1.1E-03	−1.40
*ATCG00490*	*Ribulose-biphosphate carboxylases RBCL*	3.8E-08	−1.25
*Post-translational protein modification (3)*			
*AT3G13310*	*Chaperone DnaJ-domain superfamily DJC66*	6.3E-85	3.80
*AT2G15310*	*ADP-ribosylation factor ARFB1A*	5.5E-07	2.02
*AT5G10770*	*Eukaryotic aspartyl protease family protein*	1.9E-02	1.26
*Response to iron ion and indole glucosinolate metabolic process (1)*			
*AT4G31940*	*Cytochrome P450 CYP82C4*	7.7E-03	4.46
*Systemic acquired resistance (1)*			
*AT1G75040*	*Pathogenesis-related gene 5, PR5*	1.3E-03	3.69
*Response to abscisic acid (1)*			
*AT5G27420*	*Carbon/nitrogen insensitive ubiquitin ligase CNI1*	2.9E-02	2.22
*Auxin response, polar transport and activated-signaling (1)*			
*AT1G29460*	*SAUR-like auxin responsive SAUR65*	4.3E-02	−1.26
*Miscellaneous (4)*			
*AT1G10140*	*Uncharacterized conserved protein*	9.2E-03	1.50
*AT1G78450*	*SOUL-heme binding protein*	5.9E-04	1.43
*AT2G40095*	*Alpha/beta hydrolase related protein*	4.2E-02	1.36
*AT2G07706*	*Hypothetical protein*	9.2E-05	−1.53

^a^*FDR* False Discovery Rate

The differentially expressed genes (DEGs) with increased transcript abundance in the melatonin (100 μM) treatment included two plant defensin genes *AT2G26010* (*PDF 1.3*) and *AT2G26020* (*PDF 1.2 b*) which were 25-fold and 20-fold higher as compared to the control. Moreover, three *defensin-like* (DEFL) gene transcript levels were also significantly enhanced, i.e. *AT3G59930, AT5G33355* and *AT1G34047* were 30-fold, 30-fold and 29-fold higher than the control. Expression levels of genes encoding transcription factors that were significantly induced upon melatonin (100 μM) treatment included *AT1G06160* (*Ethylene responsive factor AP2/ERF59*), *AT3G51910* (*Heat shock transcription factor A7A HSFA7A* and *AT5G07100* (*WRKY DNA-binding protein26*, *WRKY26*) which were 5.5-fold, 2.9-fold and 2.3-fold higher than the control. Interestingly, among the twelve genes with decreased expression upon melatonin treatment (100 μM), seven of them were involved in the light-dependent reactions of photosynthesis such as *ATCG00330* (*Chloroplast ribosomal protein S14*, *RPS14*), ATCG00020 (*Photosystem II reaction center protein A*, *PSBA*), *ATCG00340* (*Photosystem I, PsaA/PsaB protein*, *PSAB*) with fold change of 0.3, 0.4, 0.4 and *ATCG00490* (*Ribulose-biphosphate carboxylases*, *RBCL*) with fold change of 0.4 as compared to the control, respectively. Among the DEGs with expression altered by melatonin, 21 genes were commonly expressed by both melatonin (100 μM) and NAA. Eighteen of these co-expressed genes were increased in abundance and were mainly classified as kinases (3) and lipid metabolic process (3) among others, while only one *AT4G04840* (*methionine-sulfoxide reductase*, *MSRB6*), was decreased in abundance in both melatonin (100 μM) and NAA treatments (Table 2). Whereas, *AT5G13170* (*senescence-associated gene*, *SAG12*) was significantly enhanced in expression by NAA but showed a decreased expression upon melatonin (100 μM) treatment and *AT2G44130* (*Kelch-domain containing F Box protein*, *KFB39*) had enhanced expression in melatonin (100 μM) treatment but decreased expression in NAA treatment (Fig. 3b and Table 2).

**Table 2:** DEGs influenced by both MT (100 μM) and NAA (4.5 μM) treatment, as compared to control

Gene ID	Annotation	MT		NAA	
		FDR^a^	Log2 (Fold Change)	FDR^a^	Log2 (Fold Change)
*Kinases (3)*					
*AT1G21240*	*Wall-associated kinase 3, WAK3*	1.1E-02	3.55	9.3E-05	3.91
*AT4G18250*	*Receptor serine/threonine kinase-like*	7.7E-03	3.43	1.5E-03	3.07
*AT1G21250*	*Wall-associated kinase 1, WAK1*	3.7E-02	1.55	1.1E-12	3.18
*Transcription factors (1)*					
*AT2G16720*	*Myb domain protein 7, MYB7*	1.9E-03	1.81	5.0E-03	1.36
*Senescence (1)*					
*AT5G13170*	*Senescence-associated gene, SAG12*	3.9E-02	−1.46	2.7E-10	2.70
*Transporter (1)*					
*AT1G15520*	*ABC transporter family, ABCG40*	5.8E-03	3.01	2.2E-04	3.00
*Oxidation-reduction process (1)*					
*AT4G04840*	*Methionine sulfoxide reductase, MSRB6*	1.0E-02	−1.36	2.5E-18	3.05
*Lipid metabolic process (3)*					
*AT3G48080*	*Alpha-beta hydrolases ABH*	1.0E-05	2.68	5.8E-10	3.16
*AT2G26400*	*Acireductone dioxygenase, ARD3*	4.4E-02	2.62	2.2E-03	2.62
*AT3G22231*	*Pathogen circadian controlled PCC1*	2.9E-02	2.95	4.6E-09	4.98
*Plant type hypersensitive response (1)*					
*AT3G50480*	*Homolog of RPW8, HR4*	1.9E-03	3.03	4.3E-02	1.70
*Induced systemic resistance (1)*					
*AT1G32960*	*Subtilase family protein, ATSBT3.3*	1.9E-02	2.90	1.7E-02	2.21
*Response to other organisms (2)*					
*AT2G14560*	*Late upregulated in response to downy mildew LURP1*	2.4E-02	2.90	3.0E-10	5.13
*AT5G25250*	*Flotillin-like protein 1, FLOT1*	1.9E-03	2.61	1.1E-3	2.18
*Regulation of phenylpropanoid metabolic process (1)*					
*AT2G44130*	*Galactose/oxidase kelch repeat protein*	4.6E-05	3.40	3.4E-2	3.06
*Response to abiotic stresses (2)*					
*AT1G14880*	*Plant cadmium resistance1, PCR1*	5.5E-03	6.55	2.4E-2	4.33
*AT4G02520*	*Glutathione-S transferase GSTF2*	1.3E-03	1.82	4.9E-5	1.80
*Miscellaneous (4)*					
*AT1G10340*	*Ankyrin repeat family protein*	1.0E-02	2.37	2.1E-2	1.7
*AT2G44480*	*Beta glucosidase 17, BGLU17*	3.3E-02	1.81	1.2E-3	1.84
*AT5G55450*	*Bifunctional inhibitor/lipid transfer, ATLTP4.4*	3.9E-04	1.47	1.0E-02	3.01
*AT5G53830*	*VQ-motif containing protein, MVQ3*	1.9E-03	1.25	4.3E-06	1.41

^a^ FDR = False Discovery Rate

### Gene ontology (GO) enrichment analysis of DEGs

**Fig. 4:**
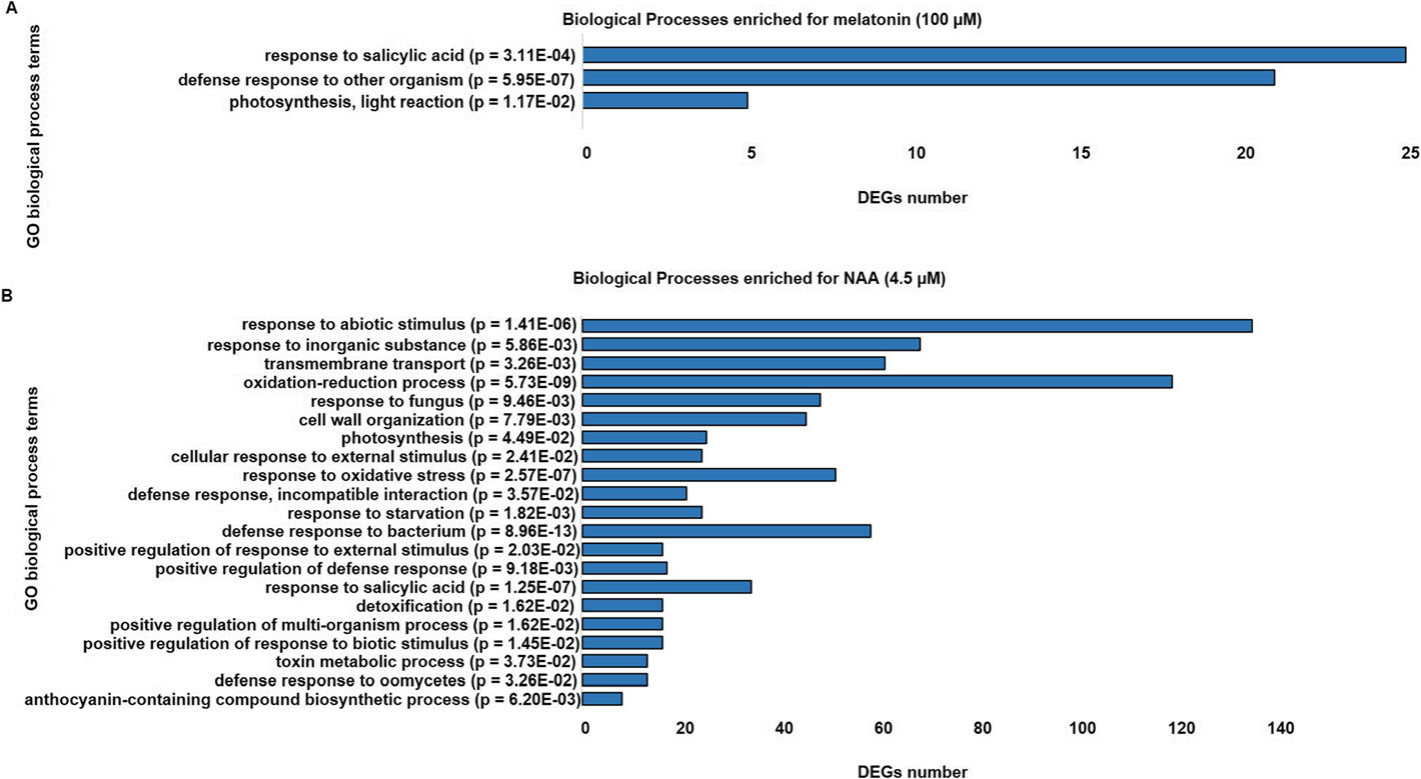
Differential GO terms in response to MT or NAA compared to untreated control. Significantly enriched GO biological process terms of (**a**) DEGs in response to MT (100 μM) and (**b**) NAA (4.5 μM) as compared to the set of all protein-coding genes in the Arabidopsis genome derived from the functional annotation classification tool in publicly available database (www.geneontology.org) [69] with *p*-values < 0.05 classified as statistically significantly different by a Fisher’s exact test with Bonferroni correction for multiple testing

Gene Ontology (GO) functional and enrichment analysis was conducted to obtain further insights into the functions of DEGs expressed by melatonin (100 μM) and NAA. DEGs that were enriched in NAA treatment were assigned 33 parent GO terms (*p* < 0.05; Bonferroni corrected). These GO terms were related to the biological processes (BP). It was evident that NAA treatment resulted in enrichment encompassing a diverse set of GO terms, based on the larger number of DEGs, while melatonin treatment led to a specific enrichment of three parent GO terms for the corresponding DEGs. Among these were “response to salicylic acid (GO:0009751)”, “defence response to other organisms (GO:0006952)” and “photosynthesis, light reaction (GO:0019864)” (Fig. 4). DEGs related to GO term “response to salicylic acid (GO:0009751)” included, among others, *AT1G75040* (*pathogenesis-related protein* 5, *PR5*), AT1G21250 (*Wall-associated receptor kinase 1*, *WAK1*), *AT1G32960* (*Subtilisin-like protease*, *SBT3.3*), *AT2G16720* (*R2R3-MYB Transcription factor, MYB7*), *AT3G50480* (*RPW8-like protein 4*). Moreover, some of the DEGs associated with enriched GO term “defence response to other organisms (GO:0006952)” included along with defensins and defensin-like genes, AT5G25250 (*Flotillin-like protein 1, FLOT1*) and *AT1G15520* (*ABC transporter G family member 40*, *ABCG40*). Further GO functional annotation of DEGs in melatonin treatment (100 μM) are detailed in Additional file 3: Table S5. GO term analysis provide further weight to the finding that while NAA exhibited a diverse response in transcriptome, the most prominent gene category induced and enriched by melatonin (100 μM) was involved in biotic stress defence and responses to hormone salicylic acid.

### Effect of melatonin treatment on expression of auxin-signalling genes

**Fig. 5:**
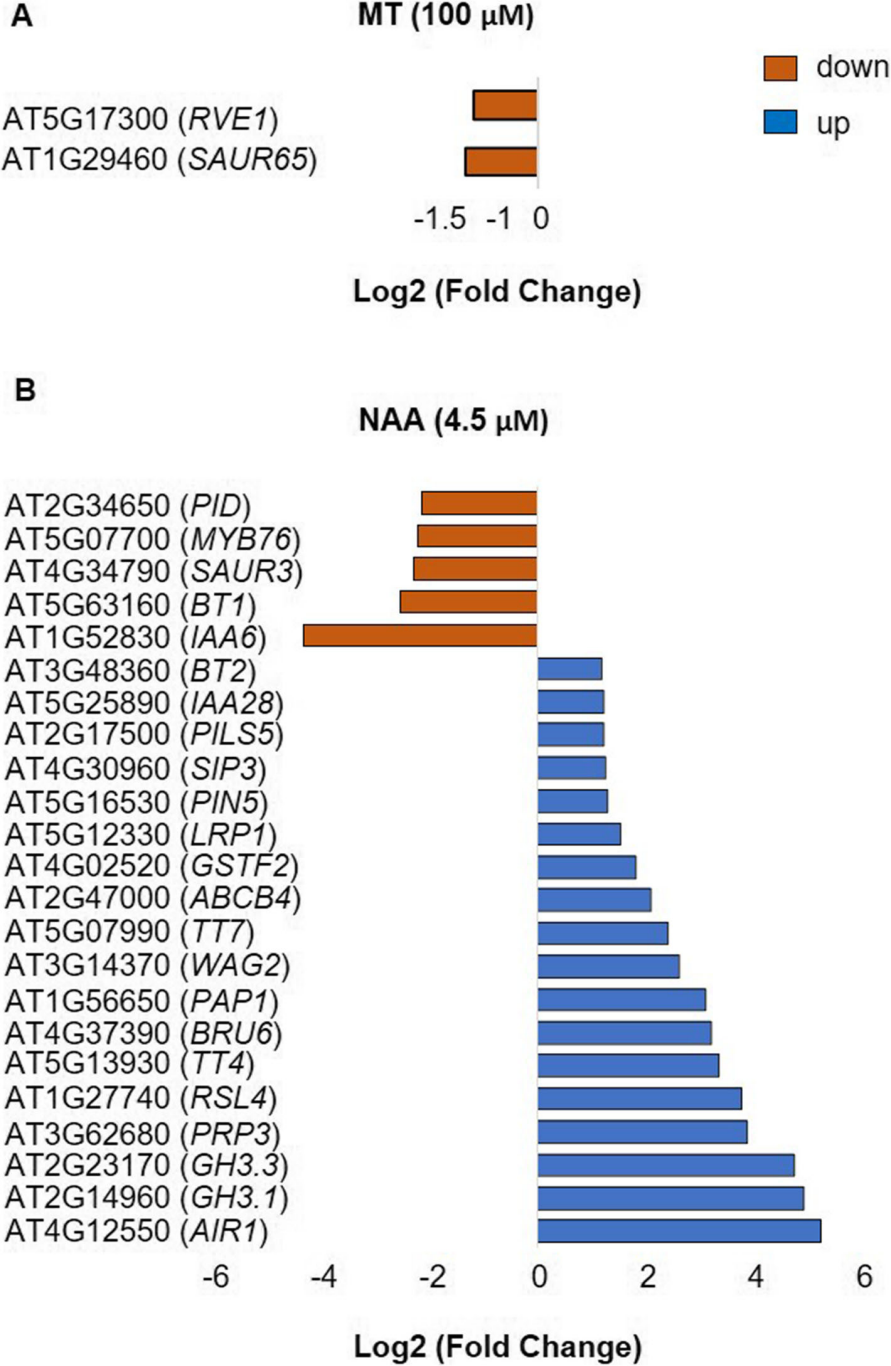
Effect of MT or NAA on DEGs involved in auxin biosynthesis, response, transport and signalling pathway. **a** DEGs of MT (100 μM) vs control involved in response to auxin, regulation of auxin biosynthetic process, polar transport and auxin activated signalling pathway. **b** DEGs of NAA (4.5 μM) vs control involved in response to auxin, auxin homeostasis, IAA amido synthetase activity, auxin polar transport, basipetal auxin transport, auxin efflux transmembrane transporter activity, auxin:proton symporter activity, auxin-activated signalling pathway, auxin biosynthetic process, auxin efflux, auxin influx and cellular response to auxin stimulus

Differentially expressed genes (DEGs) in response to melatonin-treated samples that had a GO annotation with auxin-response were analysed. Unlike NAA treatment, no significant alteration on the transcript levels of genes was observed with melatonin treatment (100 μM) in the “auxin homeostasis (GO:0010252)”, “indole-3-acetic acid amido synthetase activity (GO:0010279)”, “basipetal auxin transport (GO:0010540)”, “auxin efflux transmembrane transporter activity (GO:0010329)”, “auxin: proton symporter activity (GO:0009672)”, “auxin efflux (GO:0010315)”, “auxin influx (GO:0060919)” and “cellular response to auxin stimulus (GO: 0071365)” (Fig. 5a). However, the gene expression of *AT1G29460* (*Small Auxin Up Regulated SAUR65*) was markedly decreased 0.4-fold by melatonin compared to control. *AT1G29460* has assigned GO terms of “response to auxin (GO:0009733)”, “auxin polar transport (GO:0060918)” and “auxin-activated signalling pathway (GO:0009734)”. Additionally, expression of *AT5G17300* (*Myb-like transcription factor*, *RVE1*) was also significantly decreased by melatonin. This gene is involved in growth of hypocotyls by regulating levels of auxin dependent on circadian rhythm. It is also involved in “regulation of auxin biosynthetic process (GO:0010600)” and “auxin-activated signalling pathway (GO:0009734)” (Fig. 5). However, expression of both genes was not significantly altered by NAA, but other auxin-signalling genes were altered (Fig. 5b). Taken together, this further shows that melatonin does not affect expression of any of the known-auxin responsive genes except for *AT1G29460* and *AT5G17300* which trend toward decreased expression.

### Expression profiling of the DEGs exclusively and commonly regulated by melatonin using publicly available microarray-based expression data

**Fig. 6:**
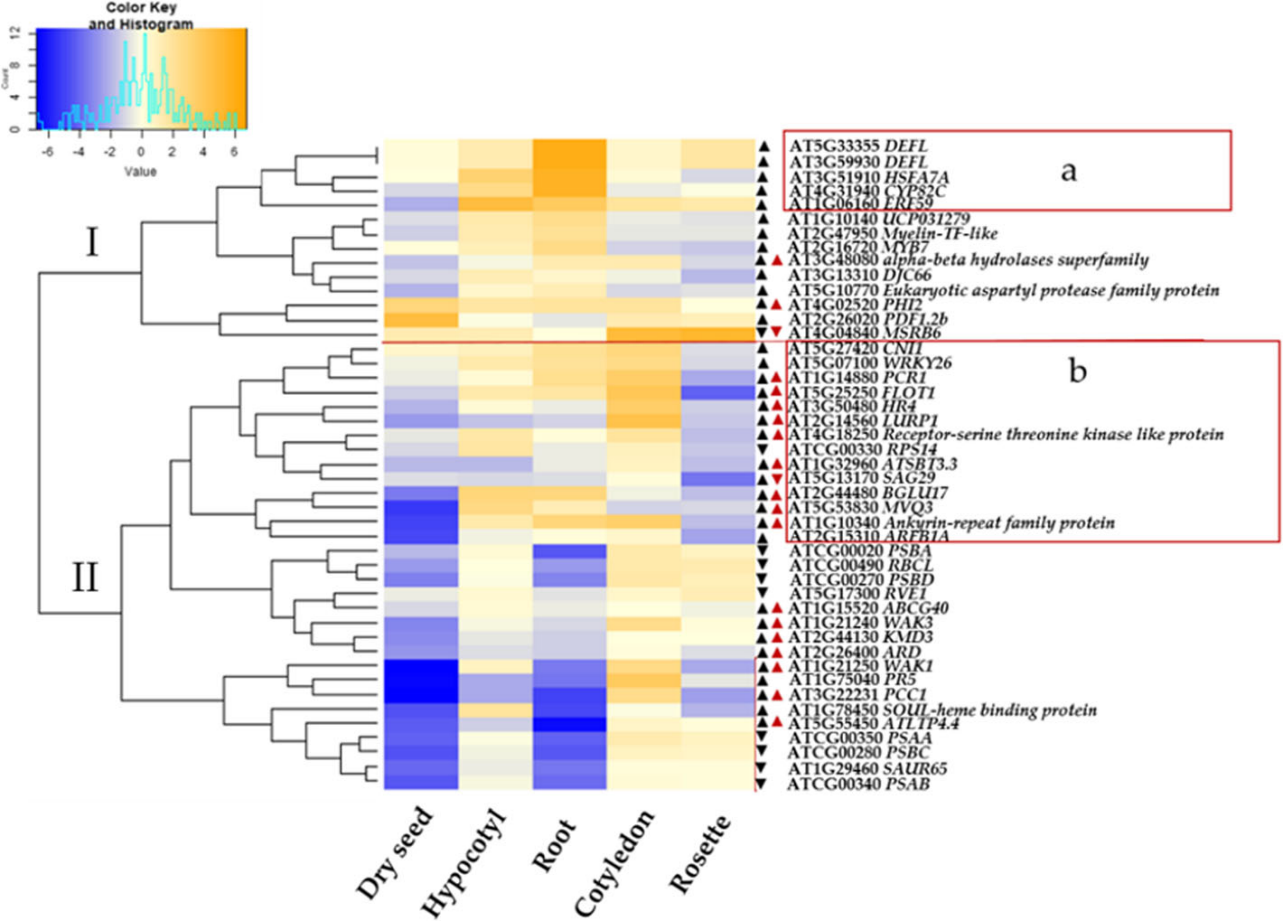
Microarray-based expression pattern of DEGs regulated by MT or commonly regulated with NAA in organs. Data were sourced from Bio-Analytic Resource (BAR). Age of *Arabidopsis* plants was 7 days cotyledon (7 days), hypocotyl (7 days), root (17 days), rosette (17 days) [71]. Arrows indicate trend of regulation (up or down) in our RNA-Seq data in response to melatonin (100 μM) (black arrows) and/or NAA (red arrows). The heat map represents hierarchical clustering of log2 transformed fold change of signal values as compared to control. All the values have been standardized prior to hierarchical clustering by z-scoring method in function ‘scale’ in R (version 3.5.2). The colour key and histogram represent the scale for relative expression with trace levels. Blue colour represents low expression levels, light represent medium expression and orange represents highest expression level. Different clusters are denoted by I and II. Boxed in red (**a** and **b**) are the DEGs forming part of each cluster

In order to obtain additional insight into the functions of DEGs regulated by melatonin we explored publicly available microarray data for Arabidopsis [70] Bio-Analytic Resource, BAR, https://bar.utoronto.ca. We focussed on selected microarray data from different organs and in response to a variety of biotic and abiotic stresses (Fig. 6 and Additional file 1: Figure S4). The data in BAR have been sourced from the previous studies (Schmid et al., 2005; Kilian et al., 2007). The genes belonged to two clusters. *AT5G33355* (*DEFL*), *AT3G59930* (*DEFL*), *AT3G51910* (*HSFA*), *AT1G06160* (*ERF59*) and *AT4G31940* (*CYP82C4)* are melatonin-unique genes that have increased transcript levels that formed a part of cluster 1(a) based on their high expression in roots (Fig. 6). Four out of these five genes are also expressed in rosettes but not to a significant level as in roots and *AT3G51910* (*HSFA*) is decreased in abundance in rosettes. Interestingly, 14 genes forming a cluster had lower expression in rosettes (cluster 2b). These included among others, *AT5G27420 (CNI1), AT5G07100 (WRKY26), AT1G14880 (PCR1)* and *AT2G15310 (ARFB1A).* Expression of 13 out of these 14 genes were shown to be highly induced in rosettes by melatonin treatment in our RNA-Seq data. Expression levels of four out of these genes have been exclusively altered by melatonin. The fact that melatonin was applied via roots in the growth medium in our experiment and is able to induce gene expression in rosettes indicates that melatonin is likely to have a systemic effect on plants.

## Discussion

Melatonin shares its biosynthetic precursor, tryptophan, with the plant hormone, auxin (indole-3-acetic acid, IAA). Owing to this, many studies have investigated if melatonin exhibits auxin-like activities. Some reports show that melatonin indeed acts in a similar manner to auxin, whilst other equally convincing reports contradict the notion [23, 36]. This has rendered the current understanding of relation between melatonin and auxin quite enigmatic. In the current study, we determined whether melatonin regulates auxin-signalling pathways in a similar fashion to auxin to get a clearer picture of the potential crosstalk between melatonin and auxin. We have found that melatonin does not act in a manner similar to auxin in *Arabidopsis thaliana* and has its own separate mode of action.

### Auxin distribution and perception is not affected by melatonin

It is known that endogenous auxin (IAA) levels are reduced by melatonin (1, 50, 100 and 200 μM) when supplemented in the growth medium in Arabidopsis by enhancing expression of Zinc Finger transcription factor *ZAT6* [72]. Interestingly in the same study, *ZAT6* showed no direct binding to TACAAT motifs in the promoter of the auxin biosynthetic gene *YUC2* as analysed by ChIP-PCR. Based on this, it is also likely that melatonin does not share AuxRE cis-regulatory elements with auxin in its signalling pathway. Our result is in accordance with previous similar studies that showed that melatonin treatment (5, 50, 100, 450 and 500 μM) was unable to affect expression of auxin-responsive marker lines *DR5: GUS* or *DII-VENUS* in *Arabidopsis* seedlings as compared to auxin (5, 10 μM IAA, 5 μM NAA) [38, 39, 73].

### Unlike auxin, melatonin does not affect mitochondrial retrograde signalling

Plants repress auxin signalling upon mitochondrial dysfunction to allocate cellular resources from growth (mediated by auxin) to stress defence response (mediated by ROS burst as a result of mitochondrial perturbation) [74]. We observed that, unlike auxin, melatonin did not suppress the induction of *AOX1a::LUC* upon Antimycin A treatment, suggesting that melatonin does not negatively regulate mitochondrial retrograde signalling and hence does not function like auxin in this pathway (Fig. 2). Moreover, melatonin also did not induce the expression of *AOX1a*. It was recently shown that rhizospheric application of melatonin (10 μM) to *Medicago sativa* plants resulted in enhanced drought tolerance in leaves by reduced H_2_O_2_ and (nitric oxide) NO contents regulating antioxidant enzymes compounds related to redox and their transcripts. Transcript levels of *AOX* were not enhanced by melatonin, implying that melatonin did not affect the electron transport chain in mitochondria under drought stress. Rather, homeostasis was maintained by regulating other enzymes involved in ROS detoxification [75]. It is thus unsurprising that melatonin also did not further enhance *AOX* expression in our study. AOX has been shown to be activated at post-translation level by certain organic acids and redox-related mechanisms [76, 77]. Intermediates of TCA (Tricarboxylic acid cycle) such as 2-oxoglutarate (2-OGs) activate AOX [78]. Interestingly, 2-ODD (2-oxoglutarate-dependent dioxygenases) or ROS-related reactions transform melatonin into its catabolites [79]. It is, thus, likely that melatonin regulates AOX post-translationally, either through ROS scavenging or via its metabolites such as 2-hydroxymelatonin and cyclic-3-hydroxy melatonin. Another possibility is that melatonin directly scavenges free radicals in mitochondria under stress very efficiently and without having to alter *AOX* gene expression or activity. This could be achieved by melatonin already present in mitochondria [41] or by entry of melatonin via mitochondrial channels such as the ABC oligopeptide transporters as has been demonstrated in mammals [80].

### Differential number of genes expressed by melatonin or auxin

Though previous studies have assessed the effect of melatonin on transcriptome of Arabidopsis, there is no report of direct comparisons of melatonin with NAA (or auxin)-treated transcriptome under identical experimental conditions. Previous transcriptome analysis of melatonin-treated Arabidopsis plants (10, 50 μM and 1 mM) have resulted in a large number of differentially expressed genes (81 to 1308 genes) in [37] and (202 to 426 genes) in [73]. However, the type of tissues analysed, method and duration of melatonin exposure, could be the cause of differences in the actual number of DEGs expressed. Tissue-specific effects of melatonin action and concentration in a variety of plant species such as *Arabidopsis thaliana, Hypericum perforatum L.* and *Oryza sativa L.* have been widely studied [81, 82]. Nevertheless, in our data, the number of melatonin-treated DEGs revealed about 40% match (i.e. out of 49 genes, 18 genes matched) with other reported studies (Additional file 1: Table S1 and Table S2). RNA-Seq data revealed that while NAA (4.5 μM) had a large and a diverse effect on transcriptome, as expected, approximately equimolar concentration of melatonin (5 μM) did not result in any significant DEGs as compared to the control. However, melatonin (100 μM) had a modest effect with expression of 49 genes significantly altered (Fig. 3). Hence, the effect of melatonin at 100 μM will be discussed in detail.

### Altered expression of genes related to biotic stress defence by melatonin

The role of melatonin in conferring enhanced tolerance to a variety of plant pathogens in diverse plant species has been documented in the past few years. These include hemi-biotrophic bacterium *Pseudomonas syringae*, biotrophic bacterium *Xanthomonas oryzae* pv. *oryzae* and necrotrophic oomycete *Phytophthora infestans* [13, 14, 83, 84]. Spray application of melatonin (1 mM) and transgenic watermelon plants overexpressing melatonin biosynthetic gene *SNAT* resulted in resistance against infection by obligate biotrophic fungus powdery mildew *Podosphaera xanthii.* This was achieved by reduction in growth of hyphae and development of conidia. Further transcriptomic analysis on watermelon leaves sprayed with melatonin (1 mM) revealed alteration in gene expression related to plant defences of both types, PAMP and effector-triggered immunity [85]. Melatonin results in plant innate immunity against *Pseudomonas syringae* PstDC3000 via mitogen-activated protein kinases (MAPK) cascades in Arabidopsis [86, 87].

Melatonin is likely to have a systemic resistance response, as in our experiment, melatonin was applied to the roots through growth medium and effect on gene expression observed in rosettes. In fact, most of the DEGs regulated by melatonin are lowly expressed in rosettes based on publicly available microarray data (Fig. 5) and showed high induction upon melatonin in our experiment. It also suggests melatonin to be a mobile signal. This has been recently tested in watermelon, where melatonin results in cold-tolerance via long distance transport [88]. Moreover, melatonin has been shown to accumulate in the leaves when applied to the roots of maize seedlings [89]. Direct uptake and translocation of melatonin or serotonin from axenic roots to shoot has been demonstrated in *St. John’s wort.* Melatonin is absorbed through the root hairs and is mainly shown to be concentrated in pericycle and endodermal tissues which is distinct to the localized distribution of auxin in vasculature [90].

DEGs regulated exclusively by melatonin treatment included pathogenesis-related (*AT1G75040 PR5),* defensins (*AT2G26010 PDF1.3, AT2G26020 PDF1.2b)* and defensin-like genes (*AT3G59930, AT5G33355, AT1G34047*) (Table 1). Melatonin has been shown to act upstream of salicylic acid [15, 91]. Our result complements previous study where exogenous melatonin (10 μM) leads to a rapid induction (within 30 min) of a variety of pathogenesis-related (PR) genes and defence genes (*PR1, PDF1.2, ICS1, ACS6, GST1, APX1,* and *VSP1)* that are activated upon treatment with SA and ethylene. This results in enhanced resistance to *P. syringae Pst* DC3000 in Arabidopsis [15]. Plant defensins are antimicrobial peptides that lead to microcidal activity by interacting with and disrupting microbial membrane [92, 93]. *PDF1.2* is decreased in expression upon attack by *P. syringae* (Cluster IIA in Additional file 1: Figure S4A). The fact that it shows higher expression by melatonin treatment in our RNA-Seq data further sheds light onto the role of melatonin in inducing defensins for biotic defence*.* SA is a mobile signalling molecule in defence that upregulates a variety of defence-related compounds such as defensins and PR proteins [94]. Thus, it is also likely that melatonin triggers SA in order to induce systemic acquired resistance. In our data, only one of the defensin-like genes, *AT5G44973*, was exclusively expressed by auxin, and it was shown to be have decreased expression (Additional file 2: Table S4). Unlike melatonin, auxin has been shown to promote diseases caused by biotrophic bacterium *P. syringae* in Arabidopsis [95]. Interestingly, one of the components of basal and SA-driven defence responses is repressing auxin signalling [96]. Apart from *P. syringae*, DEGs regulated by melatonin have also been shown to be responsive to a variety of pathogens such as *Botrytis cinerea* and *P. infestans* (Fig. 5b). This is, in agreement with previous studies where melatonin leads to disease resistance to these pathogens [84, 97]. This supports the idea that melatonin elicits broad-spectrum disease resistance. However, it is also important to note that defensins are also stimulated by environmental stresses such as osmotic stress and UV [98]. Moreover, many other defence-related genes were enhanced in expression in microarray data upon abiotic stresses (Additional file 1: Figure S4B). In this regard, it is also likely that melatonin induces defensin and other defence-related genes to confer tolerance to abiotic stresses as well. Another gene, *AT4G31940* encoding for a cytochrome P450 enzyme CYP82C4, represents a key biosynthetic enzyme of a redox-active metabolite sideretin and was strongly induced by melatonin (22-fold). Its transcript level is increased during iron-deficiency conditions [99]. Interestingly, the role of melatonin in enhancing tolerance under iron-deficient condition has been recently shown [73, 100]. Sideretin is exuded by the roots into the rhizosphere upon iron-limiting conditions. It plays a role in efficient iron acquisition by mobilizing and reducing insoluble form of iron making it readily available to the plant. Targeting the iron homeostasis is an effective strategy in plant-pathogen interactions [101, 102]. It is probable that melatonin induces *AT4G31940* CYPB2C4 as part of the defence strategy against microorganisms by enhancing iron acquisition processes such as increasing iron levels locally to activate oxidative burst upon infection site or by iron sequestration resulting in deprivation of iron to microorganism.

Among the transcription factors with altered transcript levels exclusively by melatonin that were involved in biotic defence were *AT1G06160* Ethylene responsive factor *AP2/ERF59*, *AT3G51910* Heat shock transcription factor *A7A* and AT5G07100 WRKY DNA-binding protein26 *WRKY26.* Interestingly, *PDF1.2* is transcriptionally activated by AP2/ERF59 through a crosstalk between jasmonic acid and ethylene [103]. Moreover, transcription factors belonging to AP2/ERF gene family directly activate transcription of melatonin biosynthetic genes in Cassava resulting in enhanced disease resistance against *Xanthomonas* [14]. In our data, all the mentioned genes whose expression levels were exclusively altered by melatonin were also enriched in response to salicylic acid. However, some genes whose transcript levels were commonly altered by melatonin and auxin were also enriched in response to salicylic acid (Table 1). Among these were included *AT1G21250* (*Wall-associated kinase 1*, *WAK1), AT2G14560* (*Late upregulated in response to oomycete downy mildew*, *LURP1)*, *AT2G16720* (*Myb domain protein 7*, *MYB7)*, *AT3G50480* (*Homolog of RPW8, HR4)* and *AT1G15520* (*ABC transporter family*, *ABCG40)*. Cell-wall-associated kinase 1 has been shown to be induced upon salicylic acid treatment and involved in plant defence against *P. syringae* in Arabidopsis [64]. *AT2G14560 Late upregulated in response to oomycete downy mildew (LURP1)*, plays a role in basal defence against pathogenic oomycete, *Hyaloperonospora parasitica* and is dependent on recognition that is resistance (R) protein-mediated [104]. It is likely that melatonin and auxin regulate certain common defence-related processes by regulating a few similar pathways via salicylic acid. A partial crosstalk between melatonin and auxin (IAA) was observed in a study where melatonin increased IAA levels upon infection by Fusarium wilt (Foc4) in banana [83].

### Influence of melatonin on auxin-responsive gene expression signatures

The *Small-auxin-up RNA* (*SAUR*) family is among the three gene families that are known to show a rapid and transient induction upon auxin treatment [105]. They are known to have distinct expression patterns and responsiveness to auxin. Based on this, they have been classified into two sub-clades. *SAUR65* belongs to clade-I, where all *SAUR* genes show increased expression upon auxin (IAA, 5 and 10 μM) treatment [63]. *SAUR65*, however, did not show differential expression by NAA in our experiment. This is likely to be due to differences between the type of auxin, tissue studied and duration of exposure. The exact function of *SAURs* has not been widely explored. However, some studies demonstrate *SAURs* to play a role in elongating tissues mediated by auxin in Arabidopsis and also negatively affecting auxin synthesis and polar auxin transport in rice [106]. Auxins are known to induce cell wall expansion [107]. It is likely that melatonin decreases the transcript levels of a member of *SAUR* gene family to reduce cell wall expansion to limit opportunity for pathogen invasion. *RVE1* (*AT5G17300*) is a gene encoding a Myb-like transcription factor that regulates free auxin levels in a circadian-rhythm dependent way to control hypocotyl growth [108]. Effects of melatonin in promoting hypocotyl growth in lupin have been studied previously [27]. Even though hypocotyl elongation is an ‘auxin-like’ effect, our result gives a hint that melatonin is likely to induce hypocotyl growth with its own mechanism. Additionally, expression levels of the genes involved in auxin transport such as *PIN-FORMED (PIN)* were not altered by melatonin. Auxin transport positively regulates many aspects of plant growth and disease development [109]. This further corroborates that effects of melatonin on transcriptome are direct and do not require the establishment of local auxin maxima. Our result is in line with a recent similar study where melatonin or serotonin treatment (10 or 50 μM) did not affect auxin transport in *AUX1-YFP* and *PIN1/2/4/7-GFP* marker lines in Arabidopsis. However, auxin treatment as control or direct comparison for this experiment was not used [73]. However, inhibitory concentration of melatonin (600 μM) has been shown to reduce root growth and meristem by reducing the expression of auxin polar transport marker lines such as *PIN1/2/4/7-GFP.* This suggests that inhibitory effect of melatonin on root growth in Arabidopsis partially requires auxin polar transport [31]. Interestingly, melatonin has been shown to travel laterally rather than in a polar manner across cells of *St. John’s wort* which further explains why it does not regulate auxin polar transport genes [90].

In other study, RNA-Seq analysis revealed that melatonin was able to induce the expression of auxin-responsive and related genes in rice roots [35]. Similarly, melatonin application to roots in tomato also leads to higher expression of auxin-related signalling genes mediated by Nitric oxide (NO) in hypocotyl [110]. While it is likely that melatonin exhibits its effects species-dependently, it is also important to note that differences exist with regards to specificity of auxin biosynthetic pathway between plant species [111]. Moreover, melatonin has been shown to possess auxinic activity compared with auxin in a variety of monocotyledons such as wheat, barley and oats [28]. However, no report of direct comparison of monocots and dicots together in response to melatonin or auxin has been described. Therefore, it is crucial to consider direct comparison of melatonin with auxin in a study looking to decipher how both indoles work under exact same set of experimental conditions.

### Decreased expression of photosynthetic genes by melatonin to enable switch between growth and defence

Melatonin treatment exclusively decreased expression of photosynthesis-related genes such as *ATCG00490 Ribulose-biphosphate carboxylases RBCL, ATCG00270 Photosystem II reaction center protein D PSBD* and *ATCG00330 Chloroplast ribosomal protein S14; RPS14* (Table 1; Fig. 4). Our result is in agreement with previous finding on melatonin (1 mM)-treated transcriptome in Arabidopsis where photosynthesis related genes trended toward decreased expression [37]. A possible explanation could be that there is a trade-off between growth and defence. One of the many components of mounting a plant defence response, is the downregulation of photosynthesis, especially genes related to light reactions. This is done in order to avoid any superfluous costs with the general growth and carefully allocate resources to activating plant defences [112]. Interestingly, a cluster of seven photosynthetic genes with decreased expression exclusively by melatonin also showed decreased expression by *P. infestans* in public microarray data (Cluster II b, Additional file 1: Figure S4A). Upon treatment by SA and bacterial peptide, elf18, the transcription factor *AT4G36990* (Heat Shock factor protein HSF4/*TFB1*) downregulates expression of genes encoding chloroplast proteins [113]. *TFB1* is involved in pre-invasive immunity as it has been shown to be a major molecular switch in regulating transition from growth-defence. It also binds to the *cis*-element *TL1* that is enriched in the promoters of genes related to plant defence [114, 115]. Interestingly, in our study, the transcription factor highly induced (17-fold) by melatonin (100 μM) treatment is *AT2G47950* (myelin transcription factor-like protein) (Table 1). At the moment, no information exists regarding the function of this gene. However, co-expression analysis reveals that *AT4G36990* (Heat Shock factor protein HSF4/ *TFB1*) is strongly co-expressed with *AT2G47950* [116] (Additional file 1: Figure S5). Myelin-transcription factor-like protein *(AT2G47950)* and its co-expressed gene *AT4G36990 (*HSF4, Heat-shock factor-like transcription factor) may thus be important for orchestrating the response of melatonin in terms of regulating the growth-to-defence transition and are interesting candidates for further investigation.

## Conclusion

In this study, we show by a direct comparison, that melatonin does not behave like an auxin, as it does not influence auxin-specific gene expression similarly to exert its functions in Arabidopsis. The predominant effect of melatonin on transcriptome of plants is systemically affecting biotic defence-signalling and response genes. Moreover, unlike auxin, melatonin does not affect retrograde signalling in mitochondria but rather affects photosynthesis-related genes potentially as a trade-off between growth and defence. This distinction between melatonin and auxin provides clarity to the current confusion in the scientific literature regarding the role/s of phytomelatonin. It would, therefore, be interesting to determine any common regulation mechanisms of these genes in melatonin-mediated biotic stress defence. The findings in this study support the role of melatonin in plant defence and this is worthy of further investigation for improving plant protection in agriculture.

## Materials and methods

### Plant materials and chemical treatments

*AOX1a*::*LUC* in *Arabidopsis thaliana* (Col-0) was previously constructed by fusing 2Kb of promoter of *AOX1a* (accession no. *AT3G22370*) to firefly luciferase *LUC* reporter as described in detail by previous study [61]. All seeds were sterilized by vapour-phase method of chlorine gas for 3 h and then stratified immersed in 0.1% agarose filled Eppendorf tubes at 4 °C for 2 days to synchronize germination. Post-stratification seeds were directly sown on either Gamborg’s B5 media (G398, PhytoTechnology, Kansas, USA) supplemented with 3% (w/v) Sucrose and 0.75% (w/v) agar (pH 5.7) in square plates (100 X 100 mm, LabServ) or on +/− melatonin (M5250, Sigma, Castle Hill, NSW) and NAA containing (N0640, Sigma, Castle Hill, NSW) ½ Murashige and Skoog (MS) media (M0404, Sigma, Castle Hill, NSW) which was then placed in controlled environment room at 23 °C, 16 h/8 h light-dark cycle and 56% humidity with 120 μmol/m^2^/s^2^ light intensity which was provided by cool fluorescent tubes.

Melatonin or auxin (NAA) were dissolved in 100% ethanol (v/v) to obtain stock concentrations which were further diluted in the medium to give the desired final concentrations as indicated for every experiment separately. Final concentration of ethanol as a solvent was 0.1% (v/v) for all treatments with same concentration and volume of ethanol to control for any potential effect arising from the solvent. Treatments were added into autoclaved medium (cooled to 55 °C), and equal amounts of media (50 mL) were added into each plate. Antimycin A (A8674, Sigma, Castle Hill NSW) was dissolved in 100% ethanol to prepare the stock solution (50 mM) and diluted with autoclaved MilliQ water to give final concentration of 50 μM in 0.1% ethanol (v/v). D-Luciferin (LUCK, GoldBio, St Louis MO) was dissolved in autoclaved MilliQ water to give final concentration of 2.5 mM. Melatonin, Antimycin A and D-luciferin treatments were covered in aluminium foil as they are light-sensitive. All solutions were prepared fresh on the day of the experiment and those intended for spraying on plants were also supplemented with 0.01% (v/v) tween-20 (P9416, Sigma, Castle Hill, NSW) to act as a spray surfactant.

### Fluorescence microscopy

Auxin-responsive marker line (*DR5*::*GFP*) was assessed for fluorescence in response to melatonin (0 μM (ethanol control), 0.1 μM, 5 μM and 50 μM) and NAA (0.1 μM) which was used as a positive control. 5-day old seedlings growing vertically on +/− melatonin or NAA-supplemented ½ MS agar media were assessed for GFP fluorescence in primary root tips. The seedlings were gently pulled off the agar plates and place on the microscopic slide with 100 μL of ½ MS liquid media. The samples were sealed with cover slip and quickly assessed under the upright epi-fluorescence microscope (Olympus BX53, DP80). GFP filter (unit name U-FGFP) was utilised with wavelengths of excitation filter at 460–480 (nm) and an emission filter at 495–540 (nm). GFP fluorescence from 8-bit converted processed images was quantified as sum of pixels in a region of fluorescence after background subtraction by integrated density method in the publicly available Java software ImageJ version 1.52a (https://imagej.nih.gov/ij/).

### Luciferase reporter bioluminescence imaging

12-day old *AOX1a::LUC* seedlings growing on Gamborg’s B5 media were transferred to +/− melatonin (0, 5, 10, 20, 50, 100 and 200 μM) or +/− NAA 4.5 μM containing media for a further 3 days. Following that, 15-day old plants were sprayed with +/− Antimycin A 50 μM. Six hours post application of Antimycin A, plants were sprayed with D-luciferin (2.5 mM GoldBio) and dark adapted for 30 min as previously described [21]. Luminescence was measured by ChemiDoc (Bio-Rad, MP). Quantification of luminescence was conducted by integrated density method in ImageJ.

### RNA-isolation

Rosettes from 15-day old *AOX1a::LUC* Arabidopsis seedlings were flash-frozen and homogenized in liquid nitrogen. Five individual rosettes were pooled per treatment with three independent biological replicates for each treatment. Total RNA was extracted using the Spectrum™ Plant Total RNA kit (Sigma, NSW, Australia) following manufacturer’s guidelines. Prior to elution, On-column DNase I digestion (Sigma, NSW, Australia) was performed on the extracted total RNA according to manufacturer’s instructions. Spectrophotometric analysis using Nanodrop™ ND-1000 (Analytical Technologies, Australia) was conducted to check RNA quality and quantity.

### RNA-sequencing and analysis

The TruSeq stranded mRNA library kit (Illumina) was used following manufacturer’s protocol to construct twelve RNA libraries which comprised three biological replicates for each treatment (0.1% ethanol, 4.5 μM NAA, 5 μM melatonin and 100 μM melatonin). The constructed libraries were sequenced using NextSeq550 system (Illumina) as 75 bp single-end reads with an average read number of 20 million per sample. The Kallisto program was used to determine transcript abundance as transcripts per million (TPM) by pseudoalignment of reads to the Araport11 model transcriptome with a k-mer length of 31 [117, 118]. For differential gene expression analysis, the sleuth program was used which utilizes a Wald test to determine differential gene expression [117]. Genes with a false discovery rate (FDR) of < 0.05 and log_2_ fold change of at least 1.2 were classified as differentially expressed. For functional analysis, gene ontology (GO) term enrichment of differentially expressed genes was conducted from a publicly available database (www.geneontology.org) and functional GO annotations for each gene was obtained from the bulk data retrieval tool in TAIR (www.arabidopsis.org).

## Supplementary information

**Additional file 1.** Description of data: **Figure S1**: Effect of melatonin on expression of auxin-responsive marker line DR5::GFP in *Arabidopsis thaliana* primary root **Figure S2**: Effect of melatonin on expression of auxin-responsive gene *AOX1a::LUC* in rosette leaves of *Arabidopsis thaliana ***Figure S3**: Differential seed development response of wild type *AOX1a::LUC *(Col-0) toward melatonin or auxin **Table S1**. Overlap analysis of differentially expressed genes (DEGs) related to auxin-responsive GO terms **Table S2**. Overlap analysis of DEGs with previous transcriptome data sets on melatonin in Arabidopsis. **Table S3**. Summary statistics of RNA-Seq data Figure S4. Microarray-based expression pattern of unique or commonly regulated DEGs by MT under stresses.

**Additional file 2:** **Table S4**. DEGs regulated by NAA as compared to the solvent control (0.1% v/v ethanol).

**Additional file 3:** **Table S5**: GO functional annotation and additional details of genes regulated by melatonin (100 μM) as comapred to the solvent control (0.1% v/v ethanol).

## Data Availability

Microarray expression data used for hierarchical clustering and heatmap are available in the Bio-Analytic Resource, BAR, (https://bar.utoronto.ca) public database. The RNA-seq data from our study were deposited in the Gene Expression Omnibus (GEO) database at NCBI (https://www.ncbi.nlm.nih.gov/geo/) with accession number GSE134079. All other datasets supporting the conclusions of this article are included within the article (and its additional files).

## Abbreviations

AOX: Alternative oxidase
DEGs: Differentially expressed genes
DR5: Direct repeat 5
FC: Fold change
FDR: False discovery rate
GO: Gene ontology
MT: Melatonin
NAA: 1-naphthalene acetic acid
RNA-Seq: RNA sequencing
SA: Salicylic acid
