# Supplementary material for: Direct comparison of Arabidopsis gene expression reveals different responses to melatonin versus auxin
Source: BMC Plant Biol. 2019 Dec 19;19:567. doi: 10.1186/s12870-019-2158-3 (PMC6921455; doi:10.1186/s12870-019-2158-3)
Supplement: Supplementary file 1 — Additional file 1. Description of data: Figure S1: Effect of melatonin on expression of auxin-responsive marker line DR5::GFP in Arabidopsis thaliana primary root Figure S2: Effect of melatonin on expression of auxin-responsive gene AOX1a::LUC in rosette leaves of Arabidopsis thaliana Figure S3: Differential seed development response of wild type AOX1a::LUC (Col-0) toward melatonin or auxin Table S1. Overlap analysis of differentially expressed genes (DEGs) related to auxin-responsive GO terms Table S2. Overlap analysis of DEGs with previous transcriptome data sets on melatonin in Arabidopsis. Table S3. Summary statistics of RNA-Seq data Figure S4. Microarray-based expression pattern of unique or commonly regulated DEGs by MT under stresses. [file 12870_2019_2158_MOESM1_ESM.docx]

**Figure S1**. **Effect of melatonin on expression of auxin-responsive marker line *DR5::GFP* in *Arabidopsis thaliana* primary root.** GFP quantification by Integrated Density in ImageJ. Statistical differences analyzed between treatment by One-way ANOVA and Tukey post-hoc test with *p*-value <0.05. Different letters denote statistical significance. Statistics was conducted on absolute values. 10 seedlings per treatment, 1 plate per treatment. Bars represent mean integrated density of treated seedlings (n=8-10). Error bars represent standard deviation. Experiment repeated thrice on independent days with similar results.

**A**


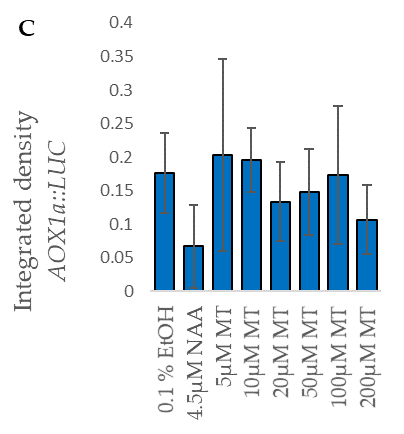


Solvent control

NAA (4.5 µM)

MT (5 µM)

MT (10 µM)

MT (20 µM)

MT (50 µM)

MT (100 µM)

MT (200 µM)


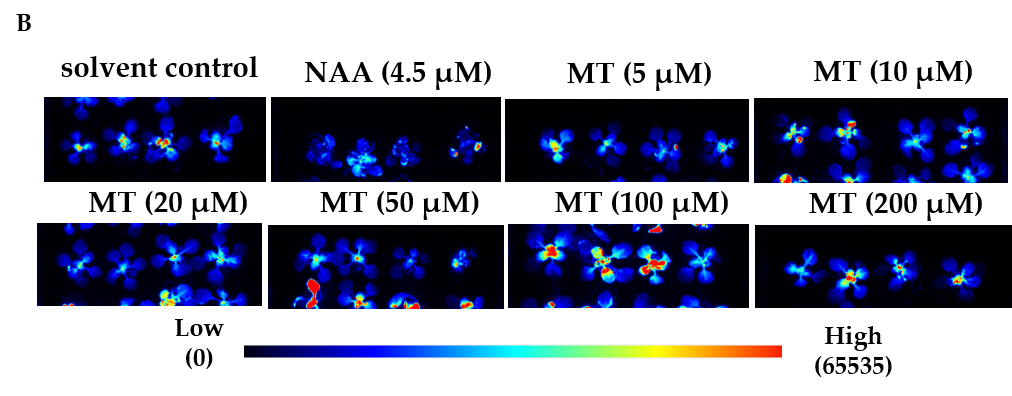


**Figure S2. Effect of melatonin on expression of auxin-responsive gene *AOX1a::LUC* in rosette leaves of *Arabidopsis thaliana*** (A) Quantification of bioluminescence of *AOX1a::LUC* +/- Antimycin A treatment by Integrated density method (ImageJ). (B) Bioluminescence images of *AOX::LUC* (wild-type Col-0) grown on +/- MT or NAA containing media for 3 days and visualized for *LUC* activity under normal growth conditions in ChemiDoc (BioRad). No statistically significant differences observed (C) Quantification of bioluminescence of *AOX1a::LUC* under normal growth conditions by Integrated density method (ImageJ). Data are expressed as mean integrated density (sum of pixels) of treated seedlings (n=12-16). Error bars represent standard deviation. Different alphabets denote statistical significance (One-way ANOVA and Tukey post-hoc), *p* < 0.05. Experiments were repeated thrice on independent days with similar results.


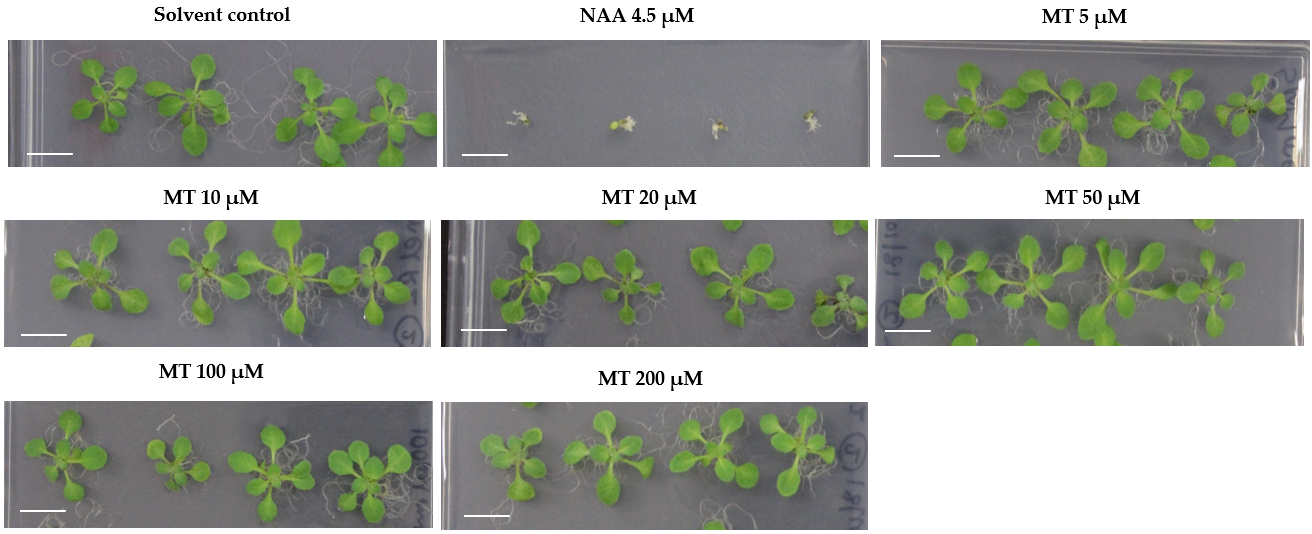


**Figure S3. Differential seed development response of wild type AOX::LUC (Col-0) toward melatonin or auxin.** Differential seed developmental responses of 14-day old *AOX1a::LUC* plants directly germinated on Gamborg’s B5 media supplemented with either melatonin or NAA. Shown are representative images. Scale bar = 1cm. Experiment was repeated thrice on independent days with similar results.

**Table S1.** **Overlap analysis of differentially expressed genes (DEGs) related to auxin-responsive GO terms**

N/A refers to transcript levels of genes not altered by the corresponding treatment

| **Gene ID** | **Our Data** | **Omelyanchuk et al., 2017 [68]**  **1 µM IAA-treated roots (6h)** |
| --- | --- | --- |
| *AT1G29460 (SAUR65)* | N/A | N/A |
| *AT5G17300 (RVE1)* | N/A | N/A |
| *AT3G48360 (BT2)* | down | up |
| *AT5G63160 (BT1)* | down | down |
| *AT1G52830 (IAA6)* | down | N/A |
| *AT2G17500 (PILS5)* | up | N/A |
| *AT2G34650 (PIID)* | up | up |
| *AT5G12330 (LRP1)* | up | N/A |
| *AT1G56650 (PAP1)* | up | N/A |
| *AT5G13930 (TT4)* | up | N/A |
| *AT1G27740 (RSL4)* | up | N/A |
| *AT2G23170 (GH3.3)* | up | up |

| **Gene ID** | **Our Data** | **Omelyanchuk et al., 2017 [68]**  **1 µM IAA-treated roots (6h)** |
| --- | --- | --- |
| *AT2G14960 (GH3.1)* | up | up |
| *AT4G12550 (AIR1)* | up | N/A |
| *AT5G16530 (PIN5)* | up | N/A |
| *AT3G14370 (WAG2)* | up | N/A |
| *AT4G37390 (GH3.4)* | up | up |
| *AT1G52830 (IAA6)* | down | N/A |
| *AT4G02520 (GSTF2)* | up | up |
| *AT3G62680 (PRP3)* | up | down |
| *AT2G47000 (ABCB4)* | up | up |
| *AT5G07990 (TT7)* | up | N/A |
| **Overlap: Approx. 40% match** |  |  |

**Table S1.** ***Contd.***

**Table S2.** **Overlap analysis of DEGs with previous transcriptome data sets on melatonin in *Arabidopsis.***

*N/A refers to transcript levels of genes not altered by the corresponding treatment*

| **Gene ID** |  | **Our Data** | **Weeda et al., 2014  [37]** | | |  | **Wan et al., 2018 [73]** |
| --- | --- | --- | --- | --- | --- | --- | --- |
|  |  |  | 100 pM MT-treated whole seedlings 16h | | 1 mM MT-treated whole seedlings 16h |  | 10 µM-MT treated whole seedlings 2 days |
| *AT3G59930* |  | up | | yes | N/A |  | up |
| *AT5G33355* |  | up | | yes | N/A |  | N/A |
| *AT1G34047* |  | up | | N/A | N/A |  | N/A |
| *AT2G26010 (PDF1.3)* |  | up | | N/A | N/A |  | N/A |
| *AT2G26020 (PDF1.2b)* |  | up | | N/A | yes |  | N/A |
| *AT2G47950* |  | up | | yes | N/A |  | N/A |
| *AT1G06160(AP2/ERF59)* |  | up | | N/A | N/A |  | N/A |
| *AT3G51910 (HSFA7A)* |  | up | | N/A | N/A |  | N/A |

**Table S2 contd.**

| **Gene ID** |  | **Our Data** | **Weeda et al., 2014  [37]** | | |  | **Wan et al., 2018 [73]** |
| --- | --- | --- | --- | --- | --- | --- | --- |
|  |  |  | 100 pM MT-treated whole seedlings 16h | | 1 mM MT-treated whole seedlings 16h |  | 10 µM-MT treated whole seedlings 2 days |
| *AT5G07100 (WRKY26)* |  | up | | N/A | N/A |  | N/A |
| *AT5G17300 (RVE1)* |  | down | | N/A | N/A |  | N/A |
| *ATCG00490 (RBCL)* |  | down | | N/A | N/A |  | N/A |
| *ATCG00270 (PSBD)* |  | down | | N/A | N/A |  | N/A |
| *ATCG00340 (PSAB)* |  | down | | N/A | N/A |  | N/A |
| *ATCG00280 (PSBC)* |  | down | | N/A | N/A |  | N/A |
| *ATCG00350 (PSAA)* |  | down | | N/A | N/A |  | N/A |
| *ATCG00020 (PSBA)* |  | down | | N/A | N/A |  | N/A |

**Table S2 contd.**

| **Gene ID** |  | **Our Data** | **Weeda et al., 2014 [37]** | | |  | **Wan et al., 2018 [73]** |
| --- | --- | --- | --- | --- | --- | --- | --- |
|  |  |  | 100 pM MT-treated whole seedlings 16h | | 1 mM MT-treated whole seedlings 16h |  | 10 µM-MT treated whole seedlings 2 days |
| *ATCG00330 (RPS14)* |  | down | | N/A | N/A |  | down |
| *AT4G31940 (CYP82C)* |  | up | | N/A | N/A |  | up |
| *AT3G13310 (DJC66)* |  | up | | N/A | N/A |  | N/A |
| *AT1G75040 (PR5)* |  | up | | N/A | N/A |  | N/A |
| *AT5G27420 (CNI1)* |  | up | | N/A | N/A |  | N/A |
| *AT2G15310 (ARFB1A)* |  | up | | N/A | N/A |  | N/A |
| *AT1G10140 (UCP031279)* |  | up | | N/A | N/A |  | N/A |
| *AT1G78450* |  | up | | N/A | N/A |  | N/A |

**Table S2 contd.**

| **Gene ID** |  | **Our Data** | **Weeda et al., 2014  [37]** | | |  | **Wan et al., 2018 [73]** |
| --- | --- | --- | --- | --- | --- | --- | --- |
|  |  |  | 100 pM MT-treated whole seedlings 16h | | 1 mM MT-treated whole seedlings 16h |  | 10 µM-MT treated whole seedlings 2 days |
| *AT2G40095* |  | up | | N/A | N/A |  | N/A |
| *AT1G29460 (SAUR65)* |  | down | | N/A | N/A |  | N/A |
| *AT1G21240 (WAK3)* |  | up | | N/A | yes |  | N/A |
| *AT4G18250* |  | up | | N/A | N/A |  | N/A |
| *AT2G16720* |  | up | | N/A | yes |  | N/A |
| *AT5G13170 (SAG29)* |  | down | | N/A | N/A |  | N/A |
| *AT1G15520 (ABCG40)* |  | up | | N/A | N/A |  | N/A |
| *AT1G14880 (PCR1)* |  | up | | N/A | yes |  | N/A |
| *AT2G44130 (KMD3)* |  | up | | N/A | yes |  | N/A |

| **Gene ID** |  | **Our Data** | **Weeda et al., 2014  [37]** | | |  | **Wan et al., 2018 [73]** |
| --- | --- | --- | --- | --- | --- | --- | --- |
|  |  |  | 100 pM MT-treated whole seedlings 16h | | 1 mM MT-treated whole seedlings 16h |  | 10 µM-MT treated whole seedlings 2 days |
| *AT3G50480 (HR4)* |  | up | | N/A | yes |  | N/A |
| *AT3G22231 (PCC1)* |  | up | | N/A | yes |  | N/A |
| *AT1G32960 (ATSBT3.3)* |  | up | | N/A | yes |  | down |
| *AT2G14560 (LURP1)* |  | up | | N/A | yes |  | N/A |
| *AT3G48080* |  | up | | N/A | N/A |  | N/A |
| *AT2G26400* |  | up | | N/A | yes |  | N/A |
| *AT5G25250 (FLOT1)* |  | up | | N/A | yes |  | N/A |
| *AT1G10340* |  | up | | N/A | yes |  | down |
| *AT4G02520 (GSTF2)* |  | up | | N/A | N/A |  | N/A |

**Table S2 contd.**

**Table S2 contd.**

| **Gene ID** |  | **Our Data** | **Weeda et al., 2014  [37]** | | |  | **Wan et al., 2018 [73]** |
| --- | --- | --- | --- | --- | --- | --- | --- |
|  |  |  | 100 pM MT-treated whole seedlings 16h | | 1 mM MT-treated whole seedlings 16h |  | 10 µM-MT treated whole seedlings 2 days |
| *AT2G44480 (BGLU17)* |  | up | | N/A | N/A |  | N/A |
| *AT5G55450 (ATLTP4.4)* |  | up | | N/A | N/A |  | N/A |
| *AT5G10770* |  | up | | N/A | N/A |  | down |
| *AT5G53830 (MVQ3)* |  | up | | N/A | N/A |  | N/A |
| *AT4G04840 (MSRB6)* |  | down | | N/A | N/A |  | N/A |
| Overlap: Approx. |  |  | |  | **31% match** |  | **10% match** |

**Table S3.** Summary statistics of RNA-Seq data

|  | Total read counts (#) | Total pseudo-aligned  (#) | Average read length (bp) | Q30 bases ratio (%) |
| --- | --- | --- | --- | --- |
| Contrep1 | 23,874,897 | 17,267,556 | 75 | 93 |
| Contrep2 | 23,772,799 | 17,562,147 | 75 | 93 |
| Contrep3 | 25,553,637 | 18,955,141 | 75 | 93 |
| MT5rep1 | 22,499,526 | 16,920,223 | 75 | 93 |
| MT5rep2 | 22,565,852 | 16,933,173 | 75 | 93 |
| MT5rep3 | 24,218,870 | 18,370,942 | 75 | 93 |
| MT100rep1 | 29,105,294 | 21,607,241 | 75 | 93 |
| MT100rep2 | 29,858,496 | 21,859,754 | 75 | 93 |
| MT100rep3 | 24,357,119 | 18,875,010 | 75 | 93 |
| NAA rep1 | 23,752,930 | 18,051,162 | 75 | 93 |
| NAA rep2 | 21,771,965 | 16,510,212 | 75 | 93 |
| NAA rep3 | 23,698,912 | 17,727,290 | 75 | 93 |


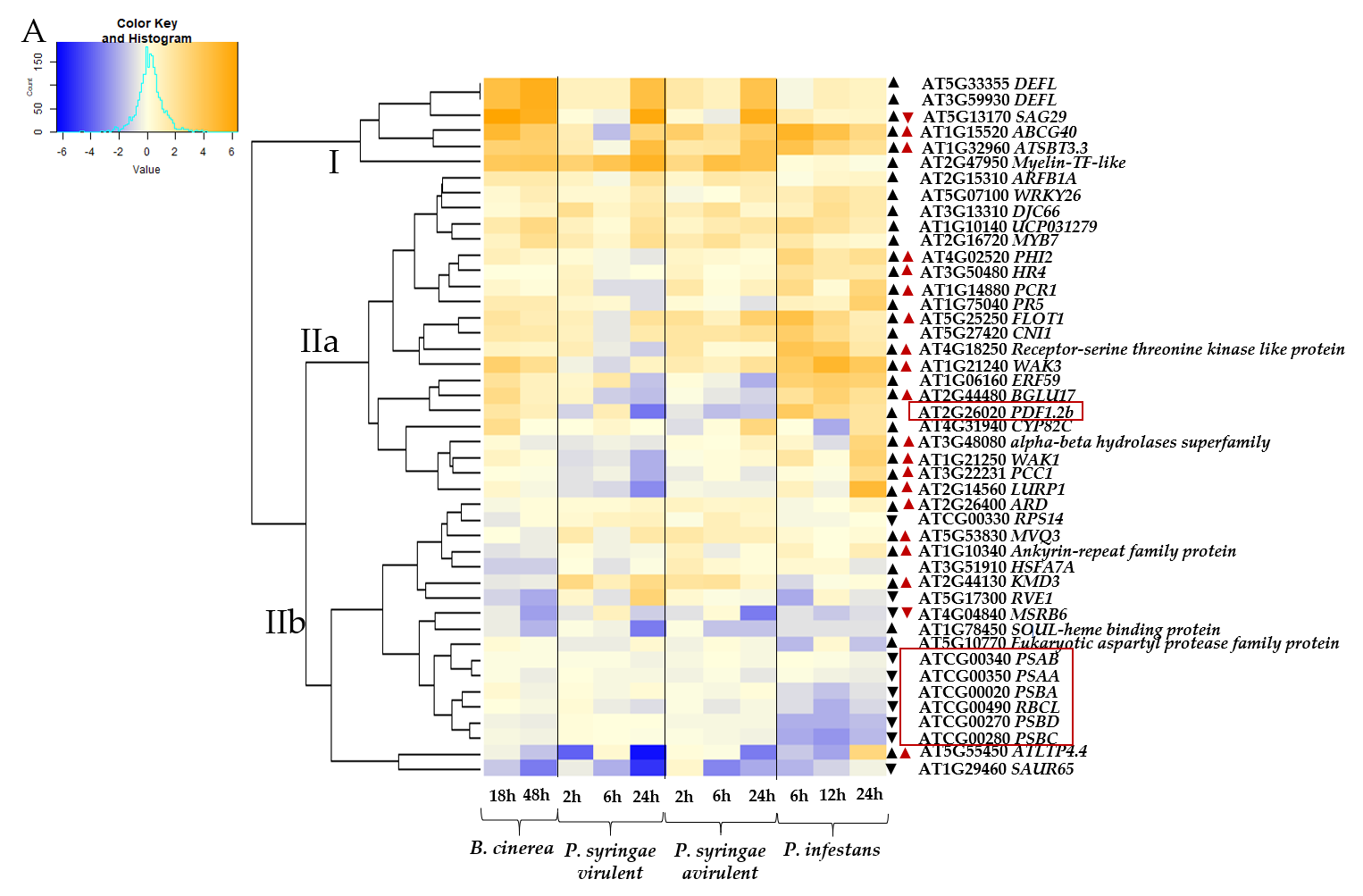


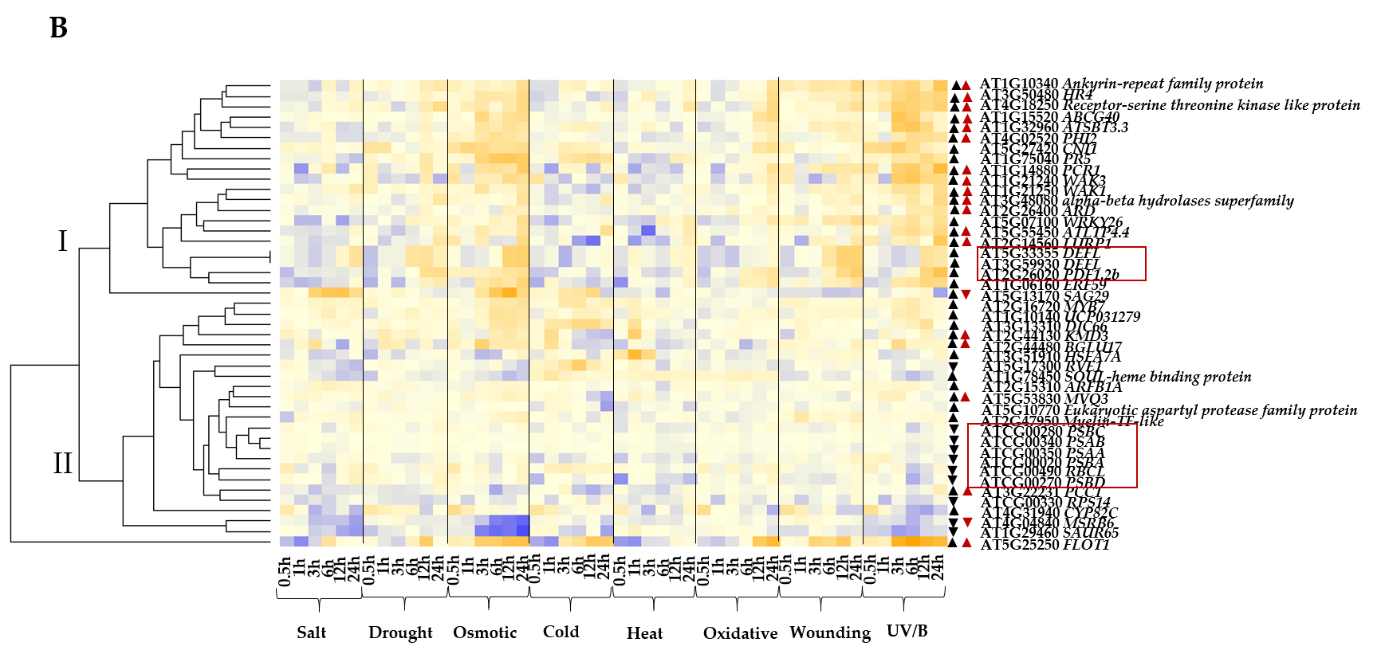


**Figure S4. Microarray-based expression pattern of unique or commonly regulated DEGs by MT under stresses.** (A) expression patterns of DEGs in 4-week old Arabidopsis leaves in different biotic conditions and (B) expression patterns of DEGs in18-day old Arabidopsis shoot in different abiotic conditions. Arrows indicate trend of regulation (up or down) in our RNA-Seq data in response to melatonin (100 µM) (black arrows) and/or NAA (red arrows). Clusters are numbered as I and II. The heat map represents hierarchical clustering of log2 transformed fold change of signal values as compared to control. Prior to clustering, the values were standardized by z-scoring method in ‘scale’ function in R (version R-3.3.2). The colour key and histogram (same for A and B) represent the scale for relative expression with trace levels. Blue colour represents low expression levels, light represent medium expression and orange represents highest expression level. Different clusters are denoted by I and II. Boxed in red are the DEGs genes forming part of each cluster and are further discussed.

**
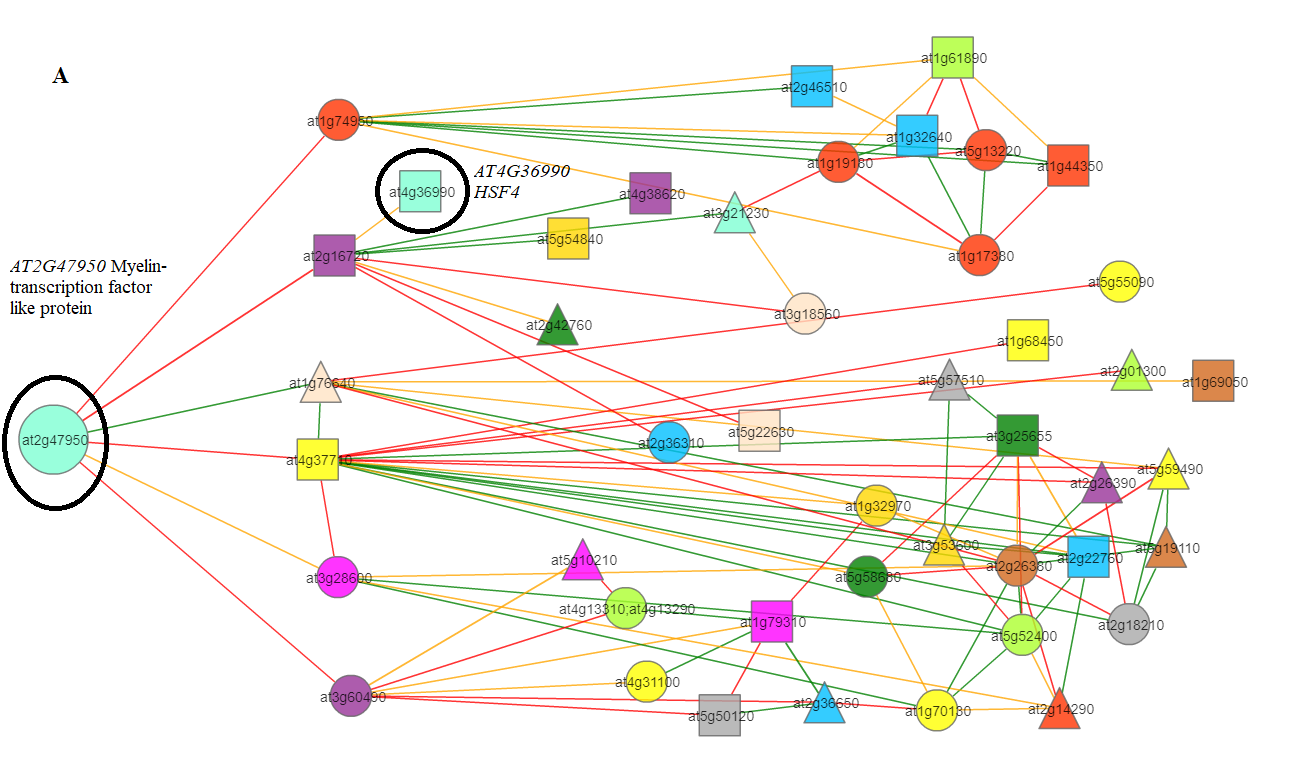
**

**B**

| **Gene ID** | **Description** |
| --- | --- |
| *AT1G74950 (TIFY10B)* | TIFY domain/Divergent CCT motif family protein |
| *AT2G16720 (MYB7)* | Encodes a member of MYB3R- and R2R3- type MYB- encoding genes |
| *AT4G36990 (HSF4)* | Heat shock transcription factor /TL1 binding transcription factor |
| *AT1G76640 (CML39)* | Calcium-binding EF-hand family protein |
| *AT4G37710 (VQ29)* | VQ motif-containing protein |
| *AT3G28600* | P-loop containing nucleoside triphosphate hydrolases superfamily protein |
| *AT3G60490 (ERF35)* | encodes a member of the DREB subfamily A-4 of ERF/AP2 transcription factor family. The protein contains one AP2 domain. There are 17 members in this subfamily including TINY. |

**Figure S5:** Co-expression network of the gene *AT2G47950* (myelin transcription factor-like protein) induced by melatonin (100 µM). Circled are *AT2G47950* and co-expressed gene *AT4G36990 (HSF4) (b)*. Genes co-expressed with *AT2G47950* in the first rank. Data are sourced from publicly available software PlaNet (<http://aranet.mpimp-golm.mpg.de/> [114].
